# Prometheus’s Fire, Part II: The Hydra of Inflammation—Multiorgan Self-Amplifying Inflammatory Loops in Obesity: Molecular Crosstalk Between Adipose Tissue, Liver, Skeletal Muscle, Pancreas and Cardiovascular System

**DOI:** 10.3390/ijms27209356

**Published:** 2026-10-21

**Authors:** Anna Grudniewska, Beata Mrozikiewicz-Rakowska, Kacper Wiszniewski, Piotr Dudek, Alina Kuryłowicz

**Affiliations:** 1 Lux Med Medical Centre in Warsaw, 49 Komitetu Obrony Robotników Street, 02-146 Warsaw, Poland; grudniewska.anna@gmail.com; 2 Department of Endocrinology, Centre of Postgraduate Medical Education, Marymoncka St. 99/103, 01-813 Warsaw, Poland; kacpor94@gmail.com (K.W.); pldudek@wp.pl (P.D.); 3 Department of General Medicine and Geriatric Cardiology, Medical Centre of Postgraduate Education, 00-401 Warsaw, Poland; alina.kurylowicz@cmkp.edu.pl

**Keywords:** adipose tissue dysfunction, NLRP3 inflammasome, metaflammation, MASLD, skeletal muscle insulin resistance, PVAT, atherosclerosis, β-cell apoptosis, obesity, self-amplifying inflammatory loops

## Abstract

Obesity should no longer be regarded as a disease confined to adipose tissue. Contemporary evidence indicates that adipose tissue dysfunction can initiate a cascade of metabolic and inflammatory responses that progressively involve multiple peripheral organs. Drawing on current molecular and translational evidence, we delineate mechanisms through which lipotoxicity, oxidative and endoplasmic reticulum stress, NLRP3 inflammasome activation and adipokine dysregulation contribute to inflammatory and metabolic dysfunction in the liver, skeletal muscle, pancreatic β-cells, vascular endothelium, perivascular adipose tissue and tumour microenvironment. Once established, organ-specific processes may become partially self-reinforcing and reciprocally amplify systemic immunometabolic dysfunction. We propose the Hydra of Inflammation as an integrative framework in which peripheral organs function not only as targets of adipose tissue-derived signals but also as secondary contributors to a progressively interconnected inflammatory network. This network-based perspective may help explain the heterogeneity of obesity-associated metabolic phenotypes and supports a shift from isolated organ-centred models towards systems-level approaches. The framework integrates the adipose tissue mechanisms discussed in Part I with the therapeutic perspectives developed in Part III of the Prometheus’s Fire series.

## 1. Introduction

Obesity is increasingly understood as a systemic immunometabolic disorder rather than a disease confined to adipose tissue. Growing evidence suggests that adipose tissue dysfunction can initiate a self-amplifying inflammatory cascade that extends across multiple organs, including the liver, skeletal muscle, pancreatic islets, cardiovascular system, perivascular adipose tissue and the tumour microenvironment. As this process develops, inflammation in these tissues may become increasingly self-sustaining, contributing to the persistence and progression of systemic metaflammation. In this context, the multi-organ nature of the inflammatory response can be viewed through the metaphor of the Hydra of Inflammation, in which several interconnected inflammatory foci may reinforce one another and maintain the broader immunometabolic disturbance [1,2].

Liver disease, skeletal muscle insulin resistance, β-cell dysfunction, cardiovascular disease and obesity-associated cancer have traditionally been studied as distinct pathological conditions. However, growing evidence points to several shared molecular processes, including lipotoxic activation of the JNK/IKKβ and NF-κB pathways, NLRP3 inflammasome activation, M1-polarised macrophage recruitment and disruption of adiponectin-mediated metabolic homeostasis. These similarities suggest that the complications of obesity may be more closely interconnected than is often reflected in organ-specific models of disease. A systems-level framework that brings these processes together is still developing and may help provide a more integrated view of obesity pathophysiology and inform the development of therapeutic strategies that target multiple components of the network.

This state-of-the-art narrative review introduces the Hydra of Inflammation as a novel integrative framework in which dysfunctional adipose tissue represents an initiating inflammatory node, while peripheral organs may become secondary sources of inflammatory signals, including cytokines, adipokines, damage-associated molecular patterns (DAMPs), and metabolic intermediates. Building on the mechanistic evidence established in Part I, The Flight of Icarus, particularly the roles of impaired adipose tissue expandability and NLRP3 inflammasome activation, this model proposes that inter-organ interactions may form self-reinforcing feedback loops that sustain metaflammation beyond the initial adipose tissue disturbance. By integrating mechanisms typically examined within individual organs into a unified systems-level model, Part II provides a broader pathophysiological basis for the therapeutic strategies discussed in Part III, The Forge of Hephaestus. The novelty of the Hydra of Inflammation concept lies not in the identification of individual inflammatory pathways, but in integrating these mechanisms into a multi-organ network of potentially self-reinforcing interactions. This framework may help explain why metabolic inflammation persists even when the initial adipose tissue disturbance is partially reduced. Where possible, mechanisms supported by experimental or clinical evidence are distinguished from those that remain predominantly hypothetical.

**Literature Search and Review Approach.** A literature search was conducted in PubMed/MEDLINE and Scopus for publications from January 2019 to August 2026, using combinations of the following search terms: adipose tissue–liver axis, MASLD, MASH, hepatic steatosis, NLRP3 inflammasome, inter-organ inflammation, feed-forward inflammatory loops, perivascular adipose tissue, PVAT, endothelial dysfunction, adipose tissue macrophages, M1/M2 polarization, adipokines, adiponectin, leptin, myeloid-derived suppressor cells, MDSC, tumor microenvironment obesity, cancer immunosurveillance, natural killer cells obesity, NKT cells, cGAS-STING, and metaflammation. Priority was given to original mechanistic studies, translational research, and high-quality systematic reviews addressing the inter-organ propagation of adipose tissue-derived inflammatory signals. Because this article is designed as a state-of-the-art narrative review, the literature was synthesized thematically rather than according to a systematic-review protocol. The selection and interpretation of evidence were guided by mechanistic relevance, methodological quality, translational significance, and thematic coherence with the integrative Hydra of Inflammation framework developed herein. The framework was used to organize and synthesize convergent evidence across tissues and organ systems rather than as a criterion for selective inclusion of individual studies. The vascular and perivascular sections draw substantially on an established body of foundational work by Heagerty and colleagues as well as on seminal mechanistic studies characterizing the role of hydrogen sulfide signaling and catecholamine handling in perivascular adipose tissue. Although these studies were published before the predefined 2019–2026 search window, they represent primary evidence underlying mechanisms that remain central to the current understanding of PVAT biology and were therefore retained. Similarly, the tumor microenvironment section incorporates landmark studies on myeloid-derived suppressor cells and natural killer cell dysfunction in obesity, which established biological mechanisms subsequently extended by more recent translational and clinical studies. PRISMA methodology was not applied because the present work is a narrative, state-of-the-art synthesis rather than a systematic review or meta-analysis. Although the primary search was restricted to publications from January 2019 onwards, foundational references predating this period were retained where they represent original mechanistic descriptions of pathways that have not been superseded by more recent literature.

The inflammatory and metabolic consequences of obesity depend not only on total adipose tissue mass but also on fat distribution, adipose tissue expandability, and adipocyte function. Visceral adiposity and ectopic lipid accumulation are generally associated with greater metabolic risk, which may partly explain differences in metabolic complications among individuals with similar BMI. Adipose tissue expandability, the capacity to recruit new adipocytes and maintain metabolic homeostasis during energy surplus, represents a key determinant of whether adipose tissue expansion remains adaptive or transitions to a lipotoxic, pro-inflammatory state.

## 2. Liver: A Key Amplifier of Inflammation in Obesity

Among all peripheral organs involved in obesity-associated metabolic disease, the liver occupies a unique position owing to its direct anatomical and functional relationship with visceral adipose tissue [3,4]. The portal circulation continuously delivers adipose tissue-derived free fatty acids (FFAs), pro-inflammatory adipokines and damage-associated molecular patterns (DAMPs) to hepatocytes and resident Kupffer cells, establishing the liver as the first peripheral organ to encounter and respond to the consequences of adipose tissue dysfunction. Crucially, however, the liver does not remain a passive target: once activated by lipotoxic and inflammatory stimuli, it generates inflammatory and metabolic signals—including IL-6, TNF-α, CRP and VLDL—that may reciprocally amplify adipose tissue dysfunction and propagate inflammation to skeletal muscle, pancreatic islets and the vasculature. Understanding hepatic immunometabolic activation is therefore essential not only for comprehending MASLD pathogenesis, but for characterizing the liver’s role as the first autonomous inflammatory amplifier within the concept of the Hydra of Inflammation.

### 2.1. Lipotoxic Activation of NF-κB and JNK in the Hepatocyte

Excessive influx of free fatty acids (FFAs) into hepatocytes, exceeding the capacity of mitochondrial β-oxidation, leads to their intracellular accumulation as triglycerides, diacylglycerol (DAG) and ceramides [5]. DAG activates the protein kinase C ε (PKCε) isoform, which phosphorylates the insulin receptor on threonine residues, thereby inhibiting downstream insulin receptor signalling and contributing to hepatic insulin resistance [5,6,7]. Simultaneously, ceramides activate the protein phosphatase PP2A, leading to dephosphorylation and inactivation of Akt kinase, which further impairs insulin signalling and activates transcription factors of the FOXO1 class, promoting gluconeogenesis. As a consequence, hepatic insulin resistance manifests as impaired suppression of gluconeogenesis, resulting in fasting hyperglycaemia despite hyperinsulinaemia, and as increased lipogenesis through constitutive activation of SREBP-1c, which further augments intrahepatic lipid accumulation and deepens lipotoxic damage, thus closing the first self-amplifying loop at the hepatic level. These lipid-derived signalling pathways ultimately converge on impaired hepatic insulin signalling, even in the presence of adequate insulin availability. This contributes to hepatic insulin resistance and promotes gluconeogenesis, de novo lipogenesis and progressive hepatic steatosis.

An independent yet equally important role in the progression of hepatic metabolic dysfunction is played by JNK1 kinase, activated by FFAs, DAG and ROS in hepatocytes [7]. JNK1 phosphorylates IRS-1 on serine residues, thereby inhibiting insulin signal transduction and additionally activating the transcription factor AP-1, which may promote transcription of pro-inflammatory genes including MCP-1, IL-8 and TNF-α. In parallel, the kinase IKKβ activates the transcription factor NF-κB, further amplifying the production of pro-inflammatory cytokines and adhesion molecules. It should be emphasised that the JNK/IKKβ/NF-κB pathway in hepatocytes constitutes a point of convergence of lipotoxic and inflammatory signals: FFAs activate JNK and IKKβ directly, through kinase signalling cascades, whilst cytokines (TNF-α, IL-6) produced by activated Kupffer cells and macrophages arriving from adipose tissue through the portal circulation activate these pathways through their respective receptors. This bidirectional stimulation creates a self-sustaining positive feedback loop in which hepatic inflammation and lipotoxicity mutually reinforce one another [7,8,9]. The hepatic feed-forward loop can be understood as a sequence of interconnected events. Excessive FFA delivery from dysfunctional visceral adipose tissue promotes hepatic lipotoxicity and insulin resistance, which can contribute to hyperglycaemia and de novo lipogenesis. These changes may further increase the availability of FFAs and promote activation of JNK, NF-κB and the NLRP3 inflammasome. Rather than following a simple linear sequence, these processes form a self-reinforcing cycle in which hepatic inflammation and metabolic dysfunction can further amplify the disturbances originating in adipose tissue. The Hydra of Inflammation therefore provides a useful conceptual framework for understanding obesity from a network medicine perspective. Within such a framework, targeting individual inflammatory mediators may be insufficient to produce sustained systemic benefits when several interconnected feedback loops remain active [10,11,12]. Selective hepatic insulin resistance may explain the coexistence of increased hepatic glucose production and enhanced de novo lipogenesis. While insulin-mediated suppression of gluconeogenesis is impaired, lipogenic pathways may remain active, partly due to hyperinsulinaemia and nutrient-dependent activation of SREBP-1c [5,13].

### 2.2. Kupffer Cells as Central Mediators of Hepatic Metaflammation

Kupffer cells, the liver’s resident macrophages, function as molecular transducers that integrate metabolic overload with chronic inflammatory signalling, positioning them as central mediators of the hepatic inflammatory loop [14]. In response to portal FFA influx and lipopolysaccharide (LPS) derived from altered intestinal microbiota, Kupffer cells undergo M1 polarisation through activation of TLR4 receptors coupled to the NF-κB pathway. Activated M1 Kupffer cells produce TNF-α, IL-1β, IL-6 and IL-12, which paracrinally damage hepatocytes and perpetuate local inflammation. In addition, activated Kupffer cells phagocytose lipid-loaded apoptotic or necroptotic hepatocytes, which in turn provides further signal for their own activation, creating an intra-hepatic self-amplifying loop. Kupffer cell activation also leads to production of TGF-β and recruitment of hepatic stellate cells, initiating the fibrogenic response that underlies MASLD progression towards MASH and cirrhosis [7,10,14,15].

TNF-α secreted by activated Kupffer cells acts paracrinally on hepatocytes, binding the TNFR1 receptor and initiating a signalling cascade that leads to activation of the JNK pathway, inhibition of IRS-1 activity and, ultimately, deepening of hepatic insulin resistance. Simultaneously, IL-6 produced by Kupffer cells activates the JAK–STAT3 pathway in hepatocytes, which under chronic conditions may promote expression of acute-phase proteins (CRP, SAA, fibrinogen)—systemic markers and mediators of obesity-related inflammation. Through the portal circulation, the liver thus becomes a major source of pro-inflammatory signals that reach adipose tissue and peripheral organs, closing the multi-organ inflammatory loop [7,8,14,16,17,18]. This bidirectional adipose tissue–liver immunometabolic crosstalk represents the molecular basis of the hepatic feed-forward loop and positions the liver as a secondary inflammatory node within the multi-organ network.

Current understanding of the role of Kupffer cells in MASLD pathogenesis has undergone substantial evolution in recent years. Two functionally distinct Kupffer cell subpopulations have been identified: one with a pro-inflammatory profile and one with an anti-inflammatory phenotype. The balance between these populations, which may be disrupted in obesity, is thought to influence the severity of hepatic inflammation and MASLD progression [14]. Furthermore, the adipose tissue–liver axis represents an important pathway for the systemic propagation of inflammation in obesity, with bidirectional crosstalk mediated through the portal circulation and systemic adipokine signalling, including leptin, adiponectin, FGF21 and chemerin [19].

Overall, these mechanisms position the liver as a key amplifier within the obesity-associated immunometabolic network (Figure 1). Portal delivery of adipose tissue-derived FFAs and inflammatory mediators promotes hepatic lipotoxicity and inflammatory signalling, contributing to impaired insulin action and MASLD progression. In turn, the liver can release systemic mediators such as IL-6 and CRP, while increased VLDL production may further contribute to metabolic and inflammatory disturbances. These signals can feed back to adipose tissue and influence other organs, including skeletal muscle, pancreatic β-cells and the vasculature. Once established, hepatic inflammation may therefore contribute to the self-reinforcing nature of obesity-associated metaflammation, while remaining interconnected with the underlying adipose tissue dysfunction [3,4,8,16,17].

Beyond their role as inflammatory targets, hepatocytes also function as active endocrine secretory cells releasing hepatokines that modulate inter-organ communication in obesity. Fibroblast growth factor 21 (FGF21), produced predominantly by the liver in response to nutritional and metabolic stress, exerts insulin-sensitising and anti-inflammatory effects in adipose tissue and skeletal muscle, and its circulating levels are elevated in obesity and non-alcoholic fatty liver disease, potentially as a compensatory response [19]. Fetuin-A, another hepatokine upregulated in hepatic steatosis, can act as an endogenous ligand for TLR4 in adipocytes and myocytes, thereby linking hepatic lipid accumulation with peripheral insulin resistance and adipose tissue inflammation [19]. Selenoprotein P, secreted by steatotic hepatocytes, has been shown to impair insulin signalling in skeletal muscle and may contribute to systemic metabolic inflexibility [19]. Together, these hepatokines establish a bi-directional communication axis between the liver and peripheral metabolic tissues that extends beyond classical inflammatory cytokine signalling and represents an additional mechanism through which hepatic dysfunction may propagate metabolic disease in obesity.

## 3. Skeletal Muscle: From Metabolic Inflexibility to Secondary Immunometabolic Amplification

Skeletal muscle plays a central role in systemic glucose homeostasis and therefore represents one of the peripheral tissues particularly susceptible to adipose tissue-derived lipotoxicity in obesity [20,21,22,23]. Responsible for approximately 80% of postprandial glucose disposal, skeletal muscle is uniquely vulnerable to FFA-mediated impairment of insulin signalling. However, the significance of skeletal muscle extends beyond its role as a metabolic target. Increasing evidence demonstrates that lipotoxicity-induced insulin resistance may transform skeletal muscle into a secondary source of pro-inflammatory signals, including IL-6, TNF-α and reactive oxygen species (ROS), that reciprocally amplify adipose tissue dysfunction and propagate metaflammation to the pancreas, vasculature and liver. Loss of metabolic flexibility represents the central functional consequence of this process and the mechanistic link between skeletal muscle lipotoxicity and systemic immunometabolic disease.

### 3.1. Lipotoxic Impairment of Insulin Signalling in the Myocyte

Ectopic lipid accumulation leads to increased levels of diacylglycerol (DAG), ceramides, and acylcarnitines in myocytes, which can interfere with insulin signal transduction. DAG activates protein kinase C theta (PKCθ), resulting in inhibitory phosphorylation of insulin receptor substrate 1 (IRS-1) and reduced interaction with the p85 regulatory subunit of phosphoinositide 3-kinase (PI3K). This impairs downstream protein kinase B (Akt) signalling and glucose transporter type 4 (GLUT4) translocation to the plasma membrane, ultimately reducing postprandial glucose uptake and contributing to skeletal muscle insulin resistance [5,6,13]. Although different lipid intermediates act through distinct signalling pathways, their effects may converge on impaired Akt-dependent GLUT4 translocation and reduced metabolic flexibility. In skeletal muscle, metabolic flexibility refers to the ability to efficiently switch between glucose and lipid oxidation according to nutritional state [20,21,22].

Skeletal muscle insulin resistance can contribute to hyperglycaemia and, over time, to the accumulation of advanced glycation end products (AGEs). AGE signalling through RAGE can activate inflammatory pathways in adipose tissue, including NF-κB signalling, providing an additional link between metabolic dysfunction in skeletal muscle and inflammation in adipose tissue [5,10]. Skeletal muscle can also contribute to this interaction through the release of myokines such as IL-6 and myostatin, while macrophage-derived TNF-α may further strengthen inflammatory signalling. Together, these processes provide a potential basis for bidirectional crosstalk between skeletal muscle and adipose tissue [8,9].

Ceramides provide an additional mechanism through which lipid accumulation can impair insulin signalling. They can reduce Akt activity by activating PP2A, which promotes Akt dephosphorylation at Thr308 and Ser473, and by enhancing PKCζ-dependent inhibitory phosphorylation of Akt. As a result, insulin signalling can remain impaired despite preserved IRS-1 tyrosine phosphorylation. This may help explain the dissociation between insulin receptor activation and the downstream metabolic response that characterises skeletal muscle insulin resistance [5,6].

Another mechanism that may contribute to persistent muscle lipotoxicity involves the accumulation of acylcarnitines, which arise from incomplete β-oxidation of long-chain fatty acids. Increased acylcarnitine levels can impair mitochondrial function and promote ROS generation, leading to activation of JNK and IKKβ in myocytes. These changes may further aggravate insulin resistance and reduce glucose oxidation, potentially favouring additional accumulation of lipid intermediates. In this way, mitochondrial dysfunction and lipid accumulation can reinforce one another and contribute to the persistence of metabolic dysfunction in skeletal muscle [5,6,8,13].

As these metabolic and inflammatory processes develop, skeletal muscle may become an important secondary component of the obesity-associated immunometabolic network. Impaired glucose uptake can contribute to postprandial hyperglycaemia, which in turn may promote β-cell stress, hepatic de novo lipogenesis and further release of FFAs into the circulation. At the same time, inflammatory signalling within skeletal muscle, including NF-κB activation and increased TNF-α and IL-6 activity, may influence adipose tissue and other peripheral organs. These reciprocal interactions place skeletal muscle within the broader network described by the Hydra of Inflammation and may contribute to the persistence of systemic metaflammation (Figure 2).

### 3.2. Skeletal Muscle as a Source and Target of Pro-Inflammatory Cytokines

Increasing evidence demonstrates that insulin-resistant myocytes acquire a pro-inflammatory secretory phenotype, releasing IL-6, TNF-α and ROS that reciprocally amplify NF-κB activity in adipocytes and Kupffer cells [20,21,22]. Skeletal muscle plays a paradoxical immunological role in the context of obesity—it may function both as a source of pro-inflammatory signals that propagate to adipose tissue and as a target of cytokines produced by adipose tissue macrophages. During acute exercise, IL-6 secreted by contracting myocytes acts in an anti-inflammatory manner—stimulating IL-10 production and inhibiting TNF-α synthesis. In obesity, however, the resting secretion of IL-6 and myostatin by myocytes with impaired insulin sensitivity acquires a pro-inflammatory character, contributing to M1 polarisation of adipose tissue macrophages and the progression of subclinical inflammation [8,15,18,24]. It is important to note that the biological effects of IL-6 depend on its source and the duration of exposure. Acute IL-6 release from contracting skeletal muscle during exercise may support metabolic adaptation and exert anti-inflammatory effects through induction of IL-10 and IL-1 receptor antagonist, whereas chronic IL-6 signalling associated with obesity-related adipose tissue dysfunction and persistent macrophage activation may contribute to metabolic inflammation and insulin resistance [4,8].

In parallel, M1 macrophages within muscle tissue secrete TNF-α, which in myocytes activates the stress kinases JNK and IKKβ. Active IKKβ phosphorylates the inhibitor IκBα, leading to nuclear translocation of the transcription factor NF-κB and increased expression of pro-inflammatory genes—including IL-6, MCP-1 and iNOS. This creates a local self-amplifying loop within skeletal muscle in which insulin resistance promotes M1 macrophage recruitment, which in turn deepens insulin resistance. Simultaneously, M1 macrophages produce IL-1β, which activates the IL-1R1 receptor on myocytes, further inhibiting insulin signalling through IRAK4–TRAF6–NF-κB pathway activation [8,10,18].

Skeletal muscle insulin resistance, by limiting postprandial glucose uptake, contributes to the perpetuation of postprandial hyperglycaemia, which constitutes a powerful secondary stimulus for β-cell hyperstimulation and IAPP overproduction in the pancreas, thereby connecting the muscle-level loop to the pancreatic self-amplifying mechanism described below. Furthermore, reduced glucose oxidation in insulin-resistant myocytes leads to augmented lipid substrate utilisation and accelerated acylcarnitine formation, closing the lipotoxic loop described in the preceding section [5,9,13]. Unlike the liver, which primarily amplifies inflammation through altered substrate metabolism and acute-phase responses, skeletal muscle propagates immunometabolic dysfunction through both metabolic—perpetuation of hyperglycaemia and lipotoxicity—and immunological mechanisms, generating cytokines that may contribute to persistence and reciprocal amplification of the multi-organ inflammatory network.

The skeletal muscle inflammatory circuit can be understood as a self-reinforcing process that begins with adipose tissue-derived lipotoxicity. Impaired insulin signalling and glucose disposal contribute to postprandial hyperglycaemia and compensatory β-cell hyperstimulation, while NF-κB-driven cytokine release from insulin-resistant muscle may further amplify adipose tissue inflammation and promote the delivery of lipotoxic signals to the liver and pancreatic islets. As this process progresses, loss of metabolic flexibility, defined as the ability to adapt substrate utilisation to nutritional state, may further compromise skeletal muscle function and contribute to persistent immunometabolic dysfunction [20,21,22,25]. Taken together, these mechanisms position skeletal muscle as both a target and a potential secondary node in the inflammatory network, linking adipose tissue-derived lipotoxicity to impaired glucose metabolism, reduced metabolic flexibility, and persistent systemic immunometabolic dysfunction. At the structural level, obesity-associated meta-inflammation in skeletal muscle is reflected by increased infiltration of CD68-positive macrophages, accumulation of crown-like structures analogous to those observed in adipose tissue, elevated intramyocellular lipid content detectable by histology and magnetic resonance spectroscopy, and reduced mitochondrial density [3,8,13]. These histological and imaging features correlate with the degree of insulin resistance and provide a morphological correlate for the molecular mechanisms discussed above.

## 4. Pancreas: From Compensatory Hyperinsulinaemia to Progressive β-Cell Inflammatory Failure

### 4.1. Lipotoxic and Glucotoxic Damage to β-Cells

Pancreatic β-cells are an important component of the obesity-associated immunometabolic network and initially compensate for peripheral insulin resistance by increasing insulin secretion. Although this response helps maintain glycaemic homeostasis, prolonged secretory demand may place increasing stress on β-cells and contribute to progressive loss of β-cell function [3,8,16]. As lipotoxic and glucotoxic stress increases, this compensatory response may become maladaptive. Elevated circulating FFAs and lipid intermediates such as ceramides, together with persistent hyperglycaemia and endoplasmic reticulum stress, increase the functional burden placed on β-cells and may promote excessive co-secretion and accumulation of islet amyloid polypeptide (IAPP, amylin). IAPP oligomerisation can promote lysosomal destabilisation and cathepsin B release, thereby contributing to NLRP3 inflammasome activation and subsequent caspase-1-dependent maturation of IL-1β and IL-18. These inflammatory signals can further impair β-cell function through autocrine and paracrine mechanisms, including IL-1 receptor 1 (IL-1R1) signalling. At the same time, activation of NF-κB and inducible nitric oxide synthase (iNOS) may further interfere with insulin gene expression and β-cell function. As β-cell mass and functional capacity decline, glycaemic control deteriorates and the secretory demand placed on the remaining β-cells increases, potentially creating conditions that favour further IAPP accumulation and amyloid formation. This reciprocal relationship between metabolic stress, IAPP accumulation and inflammasome activation provides a plausible mechanism through which pancreatic inflammation can become self-reinforcing and contribute to progressive metabolic deterioration. The proposed pancreatic feed-forward loop is summarized in Figure 3, presented later in this subsection.

Pancreatic islet β-cells are particularly sensitive to the metabolic stress associated with chronic lipotoxicity and glucotoxicity. Their limited capacity to handle excess fatty acids, together with high metabolic activity and relatively modest antioxidant defences, may contribute to this susceptibility [26]. Chronic exposure to elevated concentrations of saturated FFAs can promote intracellular lipid accumulation, including ceramides, and impair mitochondrial function, thereby increasing ROS production. Persistent oxidative stress may, in turn, place additional pressure on the endoplasmic reticulum (ER) and activate the inositol-requiring enzyme 1α (IRE1α), activating transcription factor 6 (ATF6) and protein kinase R-like endoplasmic reticulum kinase (PERK) branches of the unfolded protein response (UPR). When this response remains chronically activated, it can interfere with insulin gene expression and contribute to β-cell dysfunction and apoptosis through mechanisms involving C/EBP homologous protein (CHOP) and c-Jun N-terminal kinase (JNK) [9]. Although these pathways arise from different forms of metabolic stress, they ultimately converge on impaired insulin secretion and increasing inflammatory activity within the islet microenvironment [3,8,16].

Among saturated FFAs, palmitic acid has been particularly well studied in the context of β-cell dysfunction. In experimental models, palmitic acid can activate TLR4-dependent signalling and promote NF-κB-mediated inflammatory responses, while also interfering with ER calcium homeostasis through effects on SERCA2b. These mechanisms may converge to increase ER stress and inflammatory signalling in β-cells. By contrast, unsaturated fatty acids such as oleic acid may exert different and, in some experimental settings, less detrimental effects on β-cell function [9,26].

Glucotoxicity provides an additional source of metabolic stress. Chronic hyperglycaemia increases glucose flux through mitochondrial pathways and can enhance ROS generation, thereby promoting oxidative stress and cellular damage. Persistent hyperglycaemia may also increase the formation of advanced glycation end products (AGEs), which can activate RAGE-dependent inflammatory signalling and further increase NF-κB activity. These processes may reinforce β-cell dysfunction because declining insulin secretory capacity further compromises glycaemic control, increasing the metabolic burden placed on the remaining β-cells [9,11].

The transition from compensatory hyperinsulinaemia to β-cell dysfunction is therefore likely to reflect the gradual convergence of several forms of metabolic stress. Lipotoxicity, glucotoxicity, mitochondrial dysfunction, ER stress and inflammatory signalling may initially be partially compensated for, but their persistence can progressively reduce the ability of β-cells to maintain adequate insulin secretion. As β-cell function declines, worsening glycaemic control places further metabolic stress on the remaining β-cells, creating conditions that may reinforce the cycle of dysfunction. This pattern parallels the transition from adaptive compensation to pathological dysfunction described in Part I for adipose tissue expandability, while extending the concept to the pancreatic islet.

### 4.2. NLRP3 Inflammasome in β-Cells and the Role of IL-1β in Self-Amplification of Damage

The NLRP3 inflammasome constitutes the principal molecular mechanism through which metabolic stress is translated into chronic inflammatory destruction of pancreatic islets [3,8,16,26,27]. Islet amyloid polypeptide (IAPP, amylin), co-secreted with insulin under conditions of chronic β-cell hyperstimulation, undergoes aggregation and forms intra-islet amyloid deposits in the setting of lipotoxicity and glucotoxicity. IAPP oligomers destabilise lysosomes in β-cells and in islet-associated macrophages (IAM), triggering the release of cathepsin B into the cytoplasm and thereby activating the NLRP3 inflammasome [26,28]. Caspase-1 activated within β-cells cleaves pro-IL-1β and pro-IL-18 to their mature forms, which, binding the IL-1R1 and IL-18Rα receptors on the β-cell surface in an autocrine manner, further activate NF-κB and JNK, perpetuating the local inflammatory state. The involvement of IL-1 signalling in β-cell dysfunction has received pharmacological support from clinical studies of IL-1-targeted therapies, including anakinra and canakinumab, although the magnitude and consistency of metabolic benefits have varied across studies [18,26,28,29]. Unlike hepatic and skeletal muscle inflammation, which primarily impair insulin sensitivity, pancreatic inflammation directly compromises insulin production, a distinction that renders β-cell dysfunction a uniquely destabilising event within the Hydra of Inflammation, accelerating multi-organ metabolic deterioration by removing the principal hormonal counterbalance to systemic insulin resistance.

In parallel, resident islet macrophages (IAM), activated by FFAs, aggregated IAPP and DAMPs from apoptotic β-cells, produce IL-1β, IL-6 and TNF-α, which paracrinally reinforce the inflammatory response within the islet. Secreted IL-6 activates the JAK–STAT3 pathway in β-cells, increasing expression of the suppressor of cytokine signalling SOCS3, which inhibits insulin signalling through the JAK2 receptor, thereby creating an additional, indirect link between pancreatic inflammation and systemic insulin resistance [8,9,30].

These mechanisms have also gained support from recent clinical and translational research. Wirth et al. demonstrated that a monoclonal antibody targeting pathological IAPP aggregates reduced NLRP3 inflammasome activation and IL-1β production while preserving β-cell mass in a murine model of type 2 diabetes. These findings support the concept that pathological IAPP aggregation may represent a therapeutically relevant target in β-cell dysfunction [31].

Further support comes from recent work on the NLRP3–IL-1β pathway in type 2 diabetes. Donath et al. highlighted the context-dependent effects of IL-1β, showing that transient physiological signalling may support β-cell function, whereas persistent IL-1β exposure associated with inflammasome activation can contribute to impaired insulin expression, β-cell dysfunction and disease progression [29].

Taken together, these findings suggest that preservation of β-cell function in obesity may require attention to several interacting sources of metabolic and inflammatory stress, including lipotoxicity, glucotoxicity and NLRP3-dependent inflammation. Therapies that influence more than one of these processes may therefore provide broader protection than approaches directed at a single pathway. This concept is particularly relevant to incretin-based and metabolic therapies, whose effects extend beyond glycaemic control and may influence inflammatory and metabolic signalling within the pancreatic islet.

The non-pharmacological and pharmacological strategies that may attenuate inflammation arising from dysfunctional adipose tissue and thereby modify these interconnected pathways are discussed in detail in Part III of the Prometheus’s Fire series, The Forge of Hephaestus.

Overall, persistent peripheral insulin resistance increases the secretory demand placed on pancreatic β-cells. As this demand is sustained, increased IAPP secretion and its subsequent accumulation within the islet may promote amyloid formation and contribute to NLRP3 inflammasome activation. The resulting IL-1β-mediated inflammatory signalling can further impair β-cell function and promote β-cell loss. As insulin secretory capacity declines, glycaemic control may progressively deteriorate, increasing the metabolic burden on the remaining β-cells and reinforcing the cycle of dysfunction. In this way, pancreatic β-cell impairment can weaken the compensatory response to systemic insulin resistance and contribute to the progression from a metabolically compensated state towards overt T2DM [3,8,16,18,26,27].

## 5. Cardiovascular System: Vascular Immunometabolic Dysfunction as a Secondary Inflammatory Amplification Circuit in Obesity

The cardiovascular system represents the final major organ network recruited into obesity-associated immunometabolic inflammation. Unlike the liver or skeletal muscle, which are directly exposed to portal or systemic lipotoxic signals from adipose tissue, the vascular system occupies a unique position. It is simultaneously a target of systemic metaflammation and the anatomical conduit through which inflammatory mediators reach all other organs [10,11,12]. Once vascular immunometabolic dysfunction is established, it may be sustained and further amplified through interconnected processes involving perivascular adipose tissue (PVAT)-driven outside–in paracrine signalling to the vessel wall, endothelial inflammatory activation, and the development of an inflammatory microenvironment within atherosclerotic plaques. Together, these processes may contribute to a vascular feed-forward loop within the Hydra of Inflammation.

### 5.1. PVAT as a Paracrine Regulator of Vascular Homeostasis

Alongside well-established mechanisms such as activation of the renin–angiotensin–aldosterone system, adipokine dysregulation, expansion of circulating blood volume, increased arterial stiffness, enhanced sympathetic tone, insulin resistance, and sodium retention, chronic low-grade systemic inflammation is increasingly recognized as a key contributor to obesity-induced arterial hypertension and vascular dysfunction [32]. Among the various adipose tissue depots, systemic inflammation is also modulated by the presence of PVAT, defined as fat surrounding blood vessels in close anatomical proximity, which has emerged as a particularly relevant paracrine regulator of vascular homeostasis [33]. Through the local release of pro- and anti-inflammatory mediators, adipokines, and reactive oxygen species, PVAT can directly influence vascular tone, remodeling, and stiffness, which represent important components of the pathogenesis of arterial hypertension and subsequent atherosclerosis [33]. This PVAT-to-vessel ‘outside–in’ signalling constitutes a unique, direct pathway linking adipose tissue dysfunction to vascular inflammation that is not adequately captured by classical biomarkers of systemic inflammation such as CRP. This transition of PVAT from a physiologically anti-contractile endocrine tissue to a pro-inflammatory vascular immunometabolic organ represents a critical link between obesity-associated adipose tissue dysfunction and vascular inflammation [32,33,34,35].

Local activation of the renin–angiotensin–aldosterone system thereby establishes one of the most important positive feedback mechanisms within dysfunctional PVAT: Ang II-induced inflammation and oxidative stress further impair adiponectin secretion and promote macrophage infiltration, which in turn amplifies local RAAS activity, creating a self-reinforcing vasoconstrictive and pro-inflammatory loop [36,37,38].

In the context of obesity, hyperplasia and/or hypertrophy of adipocytes within PVAT contribute to a shift toward a pro-inflammatory and vasoconstrictive milieu. This is accompanied by infiltration and accumulation of inflammatory cells—including B- and T-lymphocytes, natural killer T (NKT) cells, and dendritic cells within the vascular wall and PVAT [35,39]. Conversely, obesity is associated with a reduction in immunosuppressive eosinophils and decreased levels of the anti-inflammatory cytokine interleukin-4 (IL-4), alongside a phenotypic shift in resident PVAT M2 macrophages toward a pro-inflammatory M1 profile [35]. As a result, there is an increase in the local production of cytokines associated with immune activation, including TNF-α, IL-17, IL-8, IL-6, and C-C motif chemokine ligand 2 (CCL2) [35].

### 5.2. Molecular Mechanisms of PVAT-Driven Vasoconstriction

The mechanisms described above drive vascular remodeling, smooth muscle cell proliferation, and local inflammation, while also modulating vascular tone through multiple pathways, thereby influencing the associated peripheral resistance [39]. Virdis et al. demonstrated that, in obese patients compared with lean controls, excess tumor necrosis factor-α (TNF-α) arising from ongoing inflammation within PVAT, likely via activation of the c-Jun N-terminal kinase (JNK) pathway, decreases the tonic production of vasodilatory nitric oxide (NO) [40]. These findings are consistent with the study by Greenstein et al., who reported that the obesity-associated loss of PVAT anticontractile function is driven by adipocyte hypertrophy, local hypoxia, and increased TNF-αR1 expression, ultimately leading to impaired endothelial nitric oxide signalling and reduced production of vasodilatory mediators such as adiponectin [34]. Conversely, Candela et al. demonstrated that macrophages present in dysfunctional PVAT contribute to a reduction in hydrogen sulfide (H_2_S) concentration within the vascular wall, which may impair endothelium-dependent vasodilation by further decreasing endothelial nitric oxide synthase (eNOS) activity and cyclic guanosine monophosphate (cGMP) production, as well as by increasing the activity of phosphodiesterases responsible for cGMP degradation [41,42,43]. The impact of reduced H_2_S availability may be amplified by its physiological vasodilatory role, mediated through intracellular acidification via the Cl^−^/HCO_3_^−^ exchanger and activation of potassium BK channels in vascular smooth muscle cells, resulting in decreased intracellular Ca^2+^ levels, inhibition of L-type Ca^2+^ channels, and membrane hyperpolarization [44,45]. Furthermore, Candela et al. also suggested that H_2_S within the vascular wall may be directly targeted by reactive species released from macrophages, thereby enhancing its consumption and consequently reducing its bioavailability.

Notably, studies by Ayala-Lopez and Withers et al. demonstrated that, under physiological conditions, PVAT-resident eosinophils and adipocytes contribute to local catecholaminergic signalling, including β_3_-adrenergic activation, which supports the secretion of vasodilatory mediators such as nitric oxide, H_2_S and adiponectin [46,47].

This process is further facilitated by organic cation transporter 3 (OCT3) expressed on PVAT adipocytes, which enables the uptake and storage of norepinephrine released from sympathetic nerve fibers while simultaneously reducing norepinephrine-mediated stimulation of α_1_-adrenergic receptors within the vascular wall [48]. Consequently, the reduction in eosinophil abundance and decreased OCT3 expression observed in obesity-related PVAT dysfunction may further contribute to impaired vasodilation and enhanced vasoconstrictor tone. Collectively, the above inflammatory mechanisms underlying PVAT dysfunction promote increased vascular tone, thereby elevating peripheral resistance and contributing to the development of arterial hypertension [49,50].

### 5.3. Local RAAS Activation in PVAT and Angiotensin II-Mediated Vascular Remodelling

Notably, adipocytes located within PVAT, beyond their effects on the local proinflammatory milieu, actively contribute to local generation of multiple components of the renin–angiotensin–aldosterone system (RAAS) [36]. Conversely, angiotensin II interacts with angiotensin type 1 and type 2 receptors expressed on PVAT adipocytes, thereby regulating adipocyte growth and differentiation, lipid metabolism, reactive oxygen species production, and, through a positive feedback mechanism, further activation of the RAAS cascade [36]. Angiotensin II additionally suppresses endothelial nitric oxide synthase (NOS3) activity while stimulating secretion of endothelin-1 and cyclooxygenase-1 (COX-1)–derived vasoconstrictor mediators, ultimately promoting small-artery remodeling and facilitating the development of systemic arterial hypertension [37,38].

As illustrated in Figure 4, PVAT dysfunction appears to evolve as a self-reinforcing process. Adipocyte hypertrophy within PVAT promotes macrophage infiltration and the release of pro-inflammatory mediators, which progressively impair vascular relaxation through several interconnected mechanisms. These include reduced nitric oxide bioavailability, disruption of hydrogen sulfide-mediated vasodilatory signalling and angiotensin II-associated vascular remodelling. Together, these changes increase peripheral vascular resistance and contribute to the maintenance of arterial hypertension [33,34,35,36,40,41]. In turn, angiotensin II may further aggravate PVAT dysfunction by promoting oxidative stress and lipogenic activity, while impaired eosinophil-associated immunoregulation, reduced OCT3 expression and altered β_3_-adrenergic signalling can weaken the remaining vasodilatory capacity of PVAT [35,36,46,47,48]. These reciprocal interactions may gradually establish a self-reinforcing state in which local vascular and immunometabolic dysfunction contributes to the persistence and progression of hypertension [32,33,35,37,39].

### 5.4. Molecular Mechanisms of Endothelial Dysfunction

Beyond its classical haemodynamic role, the vascular endothelium functions as a dynamic immunometabolic interface that integrates inflammatory, metabolic and mechanical signals originating from the circulation and the surrounding PVAT [11,12]. As a consequence of the vascular inflammatory mechanisms described above, endothelial cells (ECs) are exposed to a range of pro-inflammatory and metabolic stimuli derived from dysfunctional adipose tissue, including elevated circulating free fatty acids (FFAs), pro-inflammatory cytokines such as TNF-α, IL-6 and IL-1β, oxidized low-density lipoprotein (oxLDL), components of the renin–angiotensin–aldosterone system and adipokines such as leptin. These signals activate NF-κB in ECs and promote the expression of adhesion molecules, including ICAM-1 and VCAM-1, as well as MCP-1, E-selectin and PAI-1. At the same time, they impair endothelial nitric oxide synthase (eNOS) expression and reduce the availability of vasodilatory NO [11]. Reduced NO bioavailability not only promotes vasoconstriction but also weakens the physiological anti-inflammatory and antithrombotic properties of the endothelium. This facilitates the adhesion of monocytes and platelets to the endothelial surface and contributes to the early stages of atherosclerotic lesion development.

Metabolic disturbances can further impair endothelial NO production. Elevated FFA concentrations increase DAG formation within endothelial cells and activate protein kinase C beta (PKCβ), which can inhibit eNOS activity through phosphorylation at Thr495. In parallel, asymmetric dimethylarginine (ADMA), an endogenous inhibitor of eNOS that accumulates in insulin resistance, can further restrict NO synthesis. Together, these mechanisms provide a link between metabolic dysfunction, impaired endothelial signalling and vascular inflammation [10,11,12].

### 5.5. Endothelial Dysfunction Promotes Atherosclerotic Plaque Formation

Endothelial dysfunction facilitates the retention of LDL particles within the subendothelial space. In the presence of increased oxidative stress, these lipoproteins undergo oxidative modification and form oxLDL. OxLDL is subsequently recognised by scavenger receptors such as CD36 and SR A on monocyte-derived macrophages, promoting their transformation into foam cells and contributing to the development of the atherosclerotic plaque [12].

As the plaque develops, macrophages contribute to its inflammatory activity through the release of cytokines and matrix metalloproteinases (MMPs). MMP-1, MMP-3 and MMP-9 can degrade components of the fibrous cap and thereby increase plaque vulnerability. At the same time, NLRP3 inflammasome activation in foam cells promotes IL-1β maturation, which may stimulate smooth muscle cell migration and further support inflammatory macrophage activation. Macrophage apoptosis, partly driven by the toxic effects of intracellular lipid accumulation, contributes to the formation of the necrotic core. Cellular debris within this region provides additional DAMPs, which can further sustain local inflammation [12].

The accumulation of foam cells therefore contributes to a self-reinforcing inflammatory environment within the plaque. Macrophage-derived TNFα, IL1β, IL6 and MMPs promote the recruitment of additional inflammatory cells and maintain activation of the surrounding vascular tissue. In parallel, IL1β and TNFα can activate NFκB in endothelial cells and vascular smooth muscle cells, increasing the expression of adhesion molecules and supporting continued monocyte recruitment [11,12,18]. Rather than representing a passive accumulation of lipids, the mature atherosclerotic plaque functions as an active inflammatory microenvironment in which NLRP3 inflammasome activation, cytokine signalling and matrix degradation interact to promote plaque progression and instability. These processes may also contribute to the propagation of inflammatory signals back to adipose tissue and other components of the systemic immunometabolic network [11,12].

As illustrated in Figure 5, dysfunctional PVAT and EAT may contribute to vascular inflammation through several interconnected mechanisms. Increased production of IL-1β, TNF-α and other inflammatory mediators can activate NF-κB in endothelial cells, impair eNOS activity and reduce NO bioavailability [30,31,32,33,38]. At the same time, increased expression of endothelial adhesion molecules promotes monocyte recruitment and their subsequent accumulation and differentiation within the vascular wall, contributing to foam cell formation and local inflammatory activation [11,12]. Oxidative stress generated by dysfunctional adipose tissue may further impair endothelial antioxidant capacity, increase NO inactivation and contribute to endothelial dysfunction [32,39,40,41,42,43]. As the atherosclerotic plaque develops, its inflammatory activity may further reinforce endothelial activation through the release of cytokines and reactive oxygen species [11,12,18]. In turn, inflammatory signals originating within the plaque may influence surrounding PVAT and sustain local cytokine production and macrophage activation, particularly in the context of inflamed epicardial and perivascular adipose tissue [12,35]. These reciprocal interactions provide a mechanistic basis for a self-reinforcing vascular inflammatory circuit within the obesity-associated immunometabolic network.

Summarizing, the cardiovascular vascular immunometabolic loop begins with endothelial activation induced by adipose tissue-derived inflammatory mediators and PVAT-derived outside–in signals. Loss of endothelial nitric oxide bioavailability, NF-κB-driven adhesion molecule expression and subendothelial macrophage accumulation establish the atherosclerotic plaque as a locally self-reinforcing inflammatory microenvironment. Plaque-derived IL-1β, TNF-α and MMP activity reciprocally amplify systemic inflammation, promoting adipose tissue dysfunction and propagating the immunometabolic network to peripheral organs. Unlike the liver, skeletal muscle and pancreatic islets, in which inflammation primarily disrupts metabolic regulation, vascular inflammation directly impairs endothelial function and vascular homeostasis, contributing to the development of hypertension [10,11,12,32,33].

## 6. Cancer: The Tumour Microenvironment as an Immunometabolic Amplifier in Obesity

In contrast to the predominantly metabolic consequences of persistent inflammation in the liver and skeletal muscle, the tumour microenvironment couples inflammatory signalling with impaired antitumour immune surveillance, creating conditions that may favour tumour progression [51,52]. Obesity-associated tumours may therefore become active participants in the immunometabolic network by responding to adipose tissue-derived cytokines, adipokines and metabolic signals while generating inflammatory mediators that can further affect systemic metabolic homeostasis. The principal mechanisms linking adipose tissue dysfunction with tumour-associated immunosuppression are summarised in Figure 6. This bidirectional interaction provides a mechanistic basis for considering obesity-associated cancer as an integrated component of the immunometabolic network rather than as an isolated complication of obesity.

### 6.1. Adipose Tissue Dysfunction and the Pro-Tumorigenic Microenvironment

Obesity-associated adipose tissue dysfunction may contribute to tumorigenesis through a combination of chronic inflammation, adipokine imbalance, hyperinsulinaemia and increased IGF-1 signalling. Excess adipose tissue is also an important source of estrogen through increased aromatase activity, while elevated leptin and pro-inflammatory cytokines, together with reduced adiponectin, may further promote a pro-tumorigenic environment [53].

Importantly, obesity does not simply increase systemic inflammation but may also alter the immune microenvironment surrounding tumours. Adipose tissue-derived inflammatory signals can promote macrophage recruitment and polarization toward immunosuppressive phenotypes characterised by increased IL-10 and TGF-β production. Experimental studies have shown that these interactions may facilitate tumour growth, lymphatic dissemination and immune evasion, with IL-6, CCL20 and CCL2 signalling contributing to the recruitment and activation of tumour-supportive immune populations [53,54,55,56]. The effects appear to be context-dependent, as individual adipose-derived mediators may simultaneously promote inflammation while impairing effective antitumour immune surveillance.

Taken together, these mechanisms suggest that dysfunctional adipose tissue can influence tumour biology not only through metabolic and hormonal alterations but also by reshaping the local immune environment. The adipose tissue–tumour axis therefore represents an important component of the obesity-associated immunometabolic network, linking systemic metaflammation with impaired immune surveillance and tumour progression [51,52,53,54,55,56].

Accumulating evidence suggests that chronic inflammation associated with adipose tissue dysfunction, together with hyperinsulinaemia and persistent activation of IGF-1 signalling, contributes to the increased risk of several obesity-associated malignancies, including breast, colorectal, oral and prostate cancer. This relationship appears to be particularly relevant in metabolically unhealthy obesity, in which hypertension, dyslipidaemia or impaired glucose metabolism may further increase cancer risk [53,54,55].

Dysfunctional adipose tissue may also promote a tumour-supportive environment through altered adipokine and sex hormone signalling. Increased aromatase activity within expanded adipose depots, together with elevated TNF-α, IL-1β, IL-6 and leptin and reduced adiponectin, may contribute to tumour development and progression [53]. Importantly, chronic systemic inflammation does not necessarily translate into effective antitumour immunity. Experimental studies suggest that adipose tissue-derived inflammatory mediators and estrogens may promote macrophage phenotypes associated with immunosuppression, including increased IL-10 production [53,56]. In postmenopausal women, increased aromatase activity in expanded adipose depots enhances peripheral oestrogen production, which may promote the development and progression of hormone receptor-positive breast cancer [53]. This mechanism illustrates how adipose tissue dysfunction can influence tumour biology through both inflammatory and endocrine pathways. Importantly, the relationship between obesity and cancer risk varies substantially according to cancer type, with the strongest associations observed for endometrial, postmenopausal breast, colorectal, and renal cell carcinomas, while the mechanistic contribution of adipose tissue dysfunction relative to other obesity-related factors differs across tumour types [53].

Several experimental observations support this adipose tissue–tumour interaction. Jung et al. reported that adipocytes from obese mice promoted an immunosuppressive and pro-metastatic tumour microenvironment through interactions with M2 macrophages and lymphatic endothelial cells, facilitating lymph node metastasis [56]. Similarly, Wunderlich et al. showed that obesity-associated IL-6 signalling promoted a tumour-supportive macrophage phenotype and increased CCL20 production, thereby facilitating recruitment of CCR6-expressing lymphocytes and contributing to colorectal cancer progression [57]. Habanjar et al. further demonstrated that interactions among adipocytes, macrophages and tumour cells may support progression of ductal carcinoma in situ toward invasive breast cancer, accompanied by enhanced inflammatory and survival signalling [53].

These findings suggest that adipose tissue dysfunction may influence tumour progression not only through systemic metabolic abnormalities but also by shaping the local immune environment. Altered adipokine signalling, macrophage polarisation and chronic inflammatory activation may therefore link obesity-associated metaflammation with impaired tumour immune surveillance, establishing the adipose tissue–tumour axis as an important component of the broader immunometabolic network [51,52,53,54,55,56,57].

### 6.2. Myeloid-Derived Suppressor Cells Impair Antitumour Immunity

Among the mechanisms linking obesity with tumour progression, expansion of myeloid-derived suppressor cells (MDSCs) provides an important connection between systemic metaflammation and local immunosuppression. MDSCs comprise a heterogeneous population of immature myeloid cells that expand in the setting of chronic inflammation through altered myelopoiesis and peripheral reprogramming of circulating myeloid cells [51,52]. Their immunosuppressive activity involves several complementary pathways regulated by transcriptional networks including NF-κB, STAT1, STAT3 and STAT6. MDSCs increase the production of reactive oxygen species (ROS), inducible nitric oxide synthase (iNOS), arginase-1 (Arg-1) and indoleamine 2,3-dioxygenase (IDO), thereby limiting the function of antitumour lymphocytes [52,58].

In T cells, ROS can promote oxidative damage and apoptosis, while increased nitric oxide production together with Arg-1 activity contributes to arginine depletion and impaired T-cell proliferation [58,59]. IDO further restricts lymphocyte expansion by reducing tryptophan availability through the kynurenine pathway [60]. MDSCs may also impair B-cell responses through disruption of IL-7–STAT5 signalling, which is important for B-cell development, survival and differentiation [61].

Experimental evidence further supports a role for MDSCs in obesity-associated tumour progression. In a murine model of oral squamous cell carcinoma, Peng et al. reported that a high-fat diet accelerated tumour growth and altered the local immune microenvironment, accompanied by enhanced immunosuppressive activity of MDSCs [62]. These findings suggest that obesity may promote tumour progression not only through chronic inflammatory signalling but also by expanding myeloid populations capable of actively suppressing antitumour immunity.

### 6.3. Lipotoxic Reprogramming of Antigen-Presenting Cells and NK Cells Impairs Cancer Immunosurveillance

Obesity-associated impairment of antitumour immunity may be further influenced by excessive lipolysis and chronically elevated circulating free fatty acids (FFAs). The effects of lipid excess appear to depend on the immune cell type and tumour context, as metabolic stress may simultaneously promote inflammatory signalling and impair immune function. Nicholas et al. reported that palmitic acid activates dendritic cells through the TLR4/MyD88/NF-κB pathway, increasing reactive oxygen species (ROS) production, caspase-1 maturation and IL-1β secretion, while at the same time impairing antigen presentation [63]. These findings illustrate how metabolic stress may induce inflammatory activation without necessarily enhancing effective antitumour immunity.

A similar relationship between lipid overload and impaired immune function has been described in natural killer (NK) cells. Michelet et al. showed that exposure to elevated FFAs promotes PPAR-dependent intracellular lipid accumulation in NK cells, accompanied by metabolic reprogramming, reduced mTOR signalling and impaired glycolysis [64]. These changes may compromise the metabolic capacity required for effective NK-cell cytotoxicity. In addition, Tang et al. reported that obesity and a high-carbohydrate diet were associated with cholesterol accumulation and increased lipid peroxidation in NK cells, providing an additional mechanism of functional impairment [65]. Hypercholesterolaemia may also affect the development of other lymphocyte populations involved in tumour surveillance. Pre-clinical studies reported reduced expression of genes involved in the differentiation of haematopoietic progenitors toward NKT cells and γδ T lymphocytes in the setting of hypercholesterolaemia, suggesting a broader effect of systemic lipid excess on antitumour immune competence [66,67].

This interplay highlights the complex immune environment associated with obesity, where chronic systemic inflammatory signalling can coexist with impaired immune surveillance. Lipid-driven dysfunction of antigen-presenting cells and cytotoxic lymphocytes may represent an additional link between obesity-associated metabolic stress and tumour progression [63,64,65,66,67].

In light of the evidence discussed above, dysfunctional adipose tissue may promote tumour progression through the combined effects of chronic inflammation, MDSC expansion, macrophage polarisation and lipid-driven impairment of antitumour immune cells. These mechanisms contribute to suppression of T-cell and NK-cell activity and may facilitate tumour immune evasion. In turn, established tumours can release inflammatory and pro-cachectic mediators, including IL-6 and TNF-α, which may further impair adipose tissue function and contribute to systemic metabolic and immune dysfunction. These reciprocal interactions provide a mechanistic basis for a self-reinforcing adipose tissue–tumour axis within the obesity-associated immunometabolic network [51,52,53,54,55,56,57,58,59,60,61,62,63,64,65,66,67].

## 7. Integrated Model: The Hydra of Inflammation as a Self-Amplifying Immunometabolic Network

Traditionally, obesity-associated complications have been investigated as independent pathological entities affecting individual organs. This reductionist approach, whilst generating fundamental mechanistic insights, has failed to explain two clinically important observations: the marked heterogeneity of metabolic phenotypes among individuals with comparable adiposity, and the frequent co-occurrence and mutual aggravation of apparently unrelated complications. The evidence reviewed in the preceding sections supports a fundamentally different conceptual framework, one in which obesity complications represent not a collection of independent organ pathologies but the clinical manifestations of a single, progressively destabilising immunometabolic network. This is the most important conclusion of the present review.

The molecular mechanisms described in the preceding sections form an integrated, three-level self-amplifying system. At the primary level, dysfunctional visceral adipose tissue constitutes the initiating node, continuously generating pro-inflammatory signals (FFA, TNF-α, IL-6, leptin) and sending them through the portal and systemic circulation to peripheral organs. At the secondary level, organ-specific inflammatory loops may partially sustain and amplify local dysfunction even when the initiating adipose-derived signals are attenuated. At the tertiary level, inter-organ cross-signalling creates reciprocal coupling between these organ-specific circuits. Figure 7 illustrates this self-amplifying integrated system.

### 7.1. A Three-Level Model of Self-Amplifying Immunometabolic Dysfunction

The Hydra of Inflammation framework describes obesity-associated immunometabolic dysfunction as a process that may evolve across three interconnected levels. Dysfunctional adipose tissue may represent an early source of metabolic and inflammatory signals. These disturbances may subsequently contribute to self-amplifying processes within peripheral organs and, through reciprocal communication between tissues, facilitate the persistence and broader propagation of immunometabolic dysfunction across the system [2,3,20,25].

Despite the diversity of organ-specific responses, several molecular mechanisms recur throughout this network. These include activation of NF-κB and the NLRP3 inflammasome, changes in macrophage inflammatory phenotypes, mitochondrial dysfunction, increased reactive oxygen species (ROS) generation, and impaired adiponectin-mediated metabolic and anti-inflammatory signalling [8,10,15,16,17,18,24,54,68,69]. Their recurrence across different tissues suggests that persistent immunometabolic dysfunction may depend not only on circulating cytokines and other inter-organ mediators but also on shared intracellular mechanisms that allow metabolically stressed tissues to respond to convergent metabolic and inflammatory stimuli [8,9,18].

#### 7.1.1. Level 1. Dysfunctional Adipose Tissue as an Early Source of Inflammatory Signalling

Loss of adipose tissue expandability and progressive adipocyte hypertrophy may initiate a series of metabolic and inflammatory disturbances [20,25]. Lipotoxicity, oxidative and endoplasmic reticulum stress, mitochondrial dysfunction, NLRP3 inflammasome activation, and altered adipokine signalling may progressively transform adipose tissue into a source of free fatty acids, cytokines, adipokines, and other bioactive mediators [3,8,15,16,17,28]. These signals may influence peripheral tissues and provide an early metabolic and inflammatory context for the organ-specific processes discussed in the preceding sections.

Importantly, this framework does not imply that adipose tissue dysfunction is the sole or universal initiating event in obesity-associated immunometabolic disease. Rather, adipose tissue is considered a major early contributor whose metabolic and inflammatory signals may interact with pre-existing or subsequently developing abnormalities in other tissues [3,8,20]. This interpretation is also consistent with the heterogeneous metabolic phenotypes observed across individuals with obesity [21].

#### 7.1.2. Level 2. Organ-Specific Self-Amplifying Processes

Once metabolic and inflammatory disturbances become established within peripheral tissues, local feedback mechanisms may reinforce tissue dysfunction and reduce metabolic resilience. In the liver, increased lipid delivery, lipotoxicity, oxidative stress, and inflammatory signalling may promote hepatic lipid accumulation and impair insulin and lipid metabolism [5,7,19]. Hepatic activation of inflammatory pathways, including IKK-β/NF-κB signalling, can in turn contribute to both local and systemic insulin resistance [7]. In skeletal muscle, intracellular lipid accumulation and associated lipid intermediates may interfere with insulin signalling, mitochondrial function, and metabolic flexibility, thereby contributing to persistent insulin resistance [5,6]. In pancreatic islets, glucolipotoxic stress, IAPP aggregation, oxidative stress, and NLRP3-associated inflammatory signalling may contribute to progressive β-cell dysfunction [9,26,29]. Within the vascular compartment, interactions between dysfunctional perivascular adipose tissue (PVAT), endothelial activation, oxidative stress, and altered vascular homeostasis may contribute to the persistence of vascular dysfunction [32,33,34,35,37,40,41,49].

Although the underlying mechanisms vary between tissues, a common pattern can be observed: metabolic stress may trigger local inflammatory and cellular stress responses that further impair tissue function. These reciprocal effects may gradually reinforce metabolic dysfunction and contribute to its persistence within individual organs [5,7,9,12,19]. However, whether such processes can become functionally self-sustaining in vivo, particularly in humans, remains uncertain.

Importantly, Level 2 does not suggest that these organ-specific responses develop in isolation. As local dysfunction progresses, affected tissues may release metabolic and inflammatory mediators that influence other organs, creating opportunities for reciprocal signalling [2,19]. These inter-organ interactions form the basis of the third level of the proposed framework.

#### 7.1.3. Level 3. Inter-Organ Inflammatory and Metabolic Amplification

Signals generated within one tissue may influence the function of other metabolically active tissues, creating reciprocal interactions that may reinforce systemic metabolic and inflammatory dysfunction [2,19]. Rather than representing a single linear pathway, these interactions can be viewed as interconnected feed-forward mechanisms through which lipid, cytokine, and metabolic signals may be exchanged between tissues. Three representative mechanisms—Lipotoxic Inter-Organ Amplification, Cytokine-Mediated Inter-Organ Amplification, and Glycaemic–β-Cell Feed-Forward Amplification—illustrate how such interactions may contribute to the persistence of obesity-associated immunometabolic dysfunction [2,5,7,19].

### 7.2. Lipotoxic Inter-Organ Amplification

Excess delivery of free fatty acids (FFAs) from dysfunctional visceral adipose tissue may increase hepatic lipid flux and contribute to hepatic lipid accumulation and altered VLDL-triglyceride secretion. Increased lipid availability in skeletal muscle may promote intracellular lipid accumulation and the formation of bioactive lipid intermediates, including ceramides, which can interfere with insulin signalling and mitochondrial function. Mitochondrial stress and increased ROS generation may subsequently activate inflammatory pathways in myocytes, including NF-κB signalling. In parallel, metabolically stressed or insulin-resistant skeletal muscle may release myokines and other stress-related mediators that can influence adipose tissue function.

Through these reciprocal interactions, changes in lipid availability and signals released from metabolically stressed tissues may contribute to the persistence of metabolic dysfunction across adipose tissue, liver, and skeletal muscle. The direction and strength of these interactions are unlikely to remain constant, but may change with disease progression and the prevailing metabolic environment [3,8,16,20,21,22].

### 7.3. Cytokine-Mediated Inter-Organ Amplification

Adipose tissue-derived IL-6 and TNF-α may contribute to hepatic acute-phase responses, including increased CRP production. At the same time, systemic inflammatory mediators may promote endothelial NF-κB activation, adhesion molecule expression, and monocyte recruitment. Vascular inflammatory activity may subsequently influence adipose tissue through cytokine-, oxidative stress-, and endothelial-derived signals. Reciprocal communication between adipose and vascular tissues may therefore contribute to the persistence of inflammatory and vascular dysfunction [8,10,11,12,16,17].

This interaction should be viewed as a potential amplification mechanism rather than as evidence that adipose tissue-derived cytokines alone determine vascular inflammation. Endothelial, hepatic, immune, and metabolic signals may all contribute to the inflammatory environment, with their relative importance likely changing across disease stages.

### 7.4. Glycaemic–β-Cell Feed-Forward Amplification

Insulin resistance in skeletal muscle may contribute to impaired glucose disposal and persistent postprandial hyperglycaemia, thereby increasing the secretory demand placed on pancreatic β-cells. Prolonged exposure to metabolic stress may promote oxidative and ER stress, IAPP aggregation, and NLRP3-associated inflammatory signalling, which may contribute to progressive impairment of β-cell function. As β-cell function declines, deterioration of glycaemic control may further increase metabolic stress in adipose tissue and other peripheral organs, potentially aggravating insulin resistance and increasing the functional demand placed on β-cells [3,8,16,18,26].

This sequence represents a metabolic feed-forward mechanism linking peripheral insulin resistance with progressive β-cell stress. Although it is consistent with experimental and clinical evidence, the temporal contribution of individual tissues and the extent to which this mechanism operates as an autonomous inter-organ loop in humans remain to be established.

### 7.5. Shared Molecular Mechanisms Across the Three Levels

Although inflammatory responses retain important organ-specific features, several molecular pathways may contribute to their amplification across the three levels of the proposed framework. These include the NLRP3 inflammasome, the NF-κB signalling axis, adipokine imbalance, and the cGAS–STING pathway. Rather than functioning as independent linear pathways, these mechanisms may interact through multiple points of crosstalk, providing a molecular basis for the persistence of immunometabolic dysfunction.

The NLRP3 inflammasome represents one of the most extensively investigated shared inflammatory nodes in obesity-associated metabolic dysfunction. Activation of NLRP3 promotes caspase-1 activation and the maturation of IL-1β and IL-18, with experimental evidence implicating this pathway in adipose tissue inflammation, insulin resistance, β-cell dysfunction, and vascular injury [11,16,17,18,24,28,68,69]. However, the magnitude and tissue-specific relevance of NLRP3 activation may differ substantially between organs and disease stages. Experimental studies targeting NLRP3 or IL-1β signalling have provided evidence for its contribution to metabolic and inflammatory dysfunction in several tissues, but whether modulation of this pathway can simultaneously disrupt multiple organ-specific processes in humans remains uncertain [11,16,17,18,24,28,68,69].

NF-κB constitutes a second major convergence point. Activated by metabolic and inflammatory stimuli, including FFAs, advanced glycation end products, and IL-1β, NF-κB promotes the expression of inflammatory mediators such as TNF-α and IL-6, as well as endothelial adhesion molecules [8,10,16]. Importantly, the relationship between NF-κB and NLRP3 is not strictly unidirectional. NF-κB-dependent transcription contributes to inflammasome priming, whereas cytokines generated following inflammasome activation may subsequently reinforce NF-κB signalling. This bidirectional crosstalk provides a plausible molecular mechanism through which metabolic stress may be converted into persistent inflammatory signalling across multiple tissues [8,10,16].

Adipokine imbalance, particularly declining adiponectin and increased leptin signalling, represents a third potential amplifier. Adiponectin exerts anti-inflammatory and insulin-sensitising effects through pathways that include AMPK and other metabolic signalling networks, while also influencing mitochondrial homeostasis and inflammatory responses. Reduced adiponectin availability may therefore weaken several endogenous mechanisms that normally constrain metabolic and inflammatory stress. Conversely, leptin signalling may become increasingly dysregulated in the setting of obesity, with leptin resistance coexisting with persistent pro-inflammatory signalling [4,15,21,26,54,55]. Together, these alterations may contribute to the destabilisation of several metabolic and inflammatory processes in parallel [4,15,21,26,54,55].

The cGAS–STING pathway represents an emerging fourth molecular node linking cellular stress and innate immune activation. Mitochondrial injury and impaired mitochondrial quality control may increase the availability of cytosolic DNA, providing a potential stimulus for cGAS activation and subsequent STING–TBK1–IRF3 signalling and type I interferon responses. Experimental evidence suggests that cGAS–STING signalling may contribute to inflammatory responses associated with metabolic dysfunction, although its tissue-specific role and relative contribution to obesity-associated inflammation remain incompletely defined [1,70,71].

Potential crosstalk between cGAS–STING and NLRP3 signalling may further increase inflammatory persistence. STING-dependent signalling may influence inflammasome priming and activation, while inflammatory mediators generated downstream of inflammasome activation may in turn exacerbate cellular and mitochondrial stress [1,68,69,70,71]. Such reciprocal interactions provide a plausible mechanism by which distinct innate immune pathways could reinforce one another, although the extent to which this interaction forms a stable self-amplifying circuit in human obesity remains to be established [1,68,69,70,71].

Taken together, these pathways are unlikely to act in isolation. Instead, they may interact at multiple levels within the broader immunometabolic network, which could help explain why interventions that improve several metabolic processes at once often produce broader effects than approaches directed at a single inflammatory mediator. Incretin-based therapies and bariatric surgery, for example, are associated with improvements across a range of metabolic and inflammatory parameters [11,27,28]. Whether these benefits involve coordinated changes in NLRP3, cGAS–STING, adipokine signalling, or other inflammatory pathways, however, remains to be clarified.

Although the mechanisms described above may interact in complex, context-dependent ways, their principal features, the organs they affect, the metabolic consequences they drive, and the strength of available evidence can be summarised schematically. Table 1 provides an overview of the main inflammatory pathways discussed throughout this review.

## 8. Conceptual Advances

This review proposes a network-based view of obesity-associated immunometabolic disease. Rather than representing isolated organ complications, obesity-related inflammation may progressively involve adipose tissue, peripheral organs and their reciprocal signalling pathways. The Hydra of Inflammation provides a conceptual framework for this process, highlighting the potential contribution of organ-specific self-amplifying loops and inter-organ communication to differences in disease phenotype. Within this framework, adipokine imbalance and inflammatory signalling may represent important links between adipose tissue dysfunction and systemic metabolic complications [3,21,22].

## 9. Knowledge Gaps and Future Perspectives

Several aspects of this model remain to be established in humans, particularly the temporal sequence and relative contribution of individual organ-specific loops and the extent to which they can sustain inflammation independently. Further studies should also clarify how NLRP3, cGAS STING and adipokine signalling interact across tissues and disease stages. Although experimental studies support several mechanisms included in the Hydra of Inflammation model, their relative contribution to human obesity remains uncertain. The temporal sequence in which organ-specific loops are recruited, and the extent to which they operate as independent drivers of disease progression, have not been established in prospective human studies. Further clinical research is needed to determine whether the proposed inter-organ inflammatory feedback loops can be reliably detected, quantified and therapeutically targeted in humans with obesity [2,3,9].

Future research should therefore focus on longitudinal human studies and on identifying biomarkers that reflect network activity rather than isolated inflammatory pathways. Determining whether distinct immunometabolic network configurations can be identified in individual patients may also support more targeted therapeutic strategies.

Overall, the proposed framework shifts the perspective from isolated obesity-related complications towards an interconnected immunometabolic system. The clinical implications of this model and its relevance to multi-target metabolic therapies are explored in Part III of this series, The Forge of Hephaestus.

## Figures and Tables

**Figure 1:**
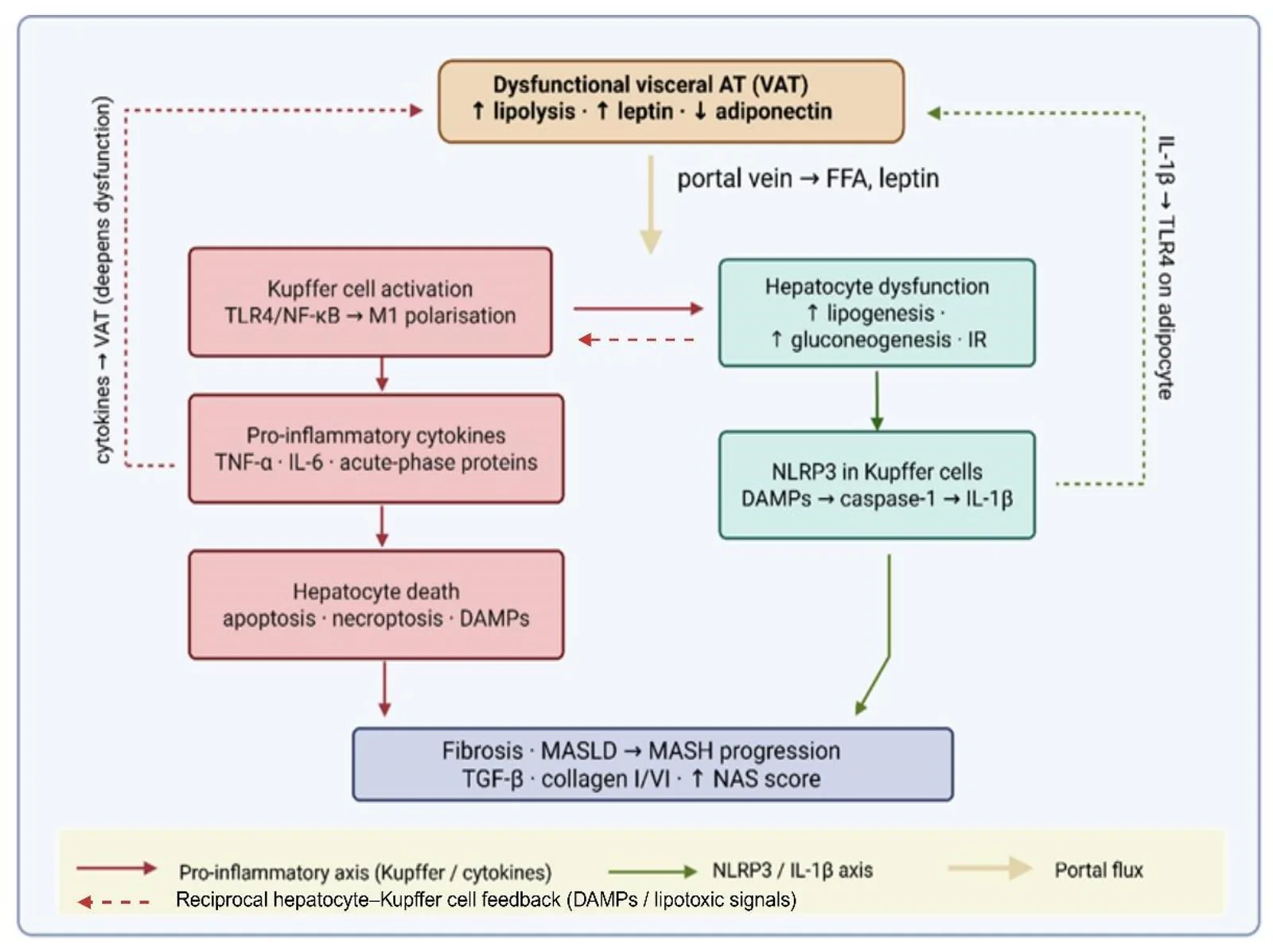
The liver as an inflammatory amplifier within the obesity-associated immunometabolic network. Visceral adipose tissue–derived signals promote hepatic inflammation and NLRP3 inflammasome activation, while hepatic inflammatory mediators may reinforce adipose tissue dysfunction, establishing a self-amplifying liver–adipose inflammatory loop. Created in BioRender. Grudniewska, A. (2026) https://BioRender.com/phbkff8. Abbreviations: AT, adipose tissue; VAT, visceral adipose tissue; FFA, free fatty acids; TLR4, Toll-like receptor 4; NF-κB, nuclear factor kappa B; TNF-α, tumor necrosis factor alpha; IL-6, interleukin-6; NLRP3, NOD-like receptor family pyrin domain containing 3; DAMPs, damage-associated molecular patterns; IL-1β, interleukin-1 beta; IR, insulin resistance; MASLD, metabolic dysfunction-associated steatotic liver disease; MASH, metabolic dysfunction-associated steatohepatitis; TGF-β, transforming growth factor beta; NAS, NAFLD Activity Score.

**Figure 2:**
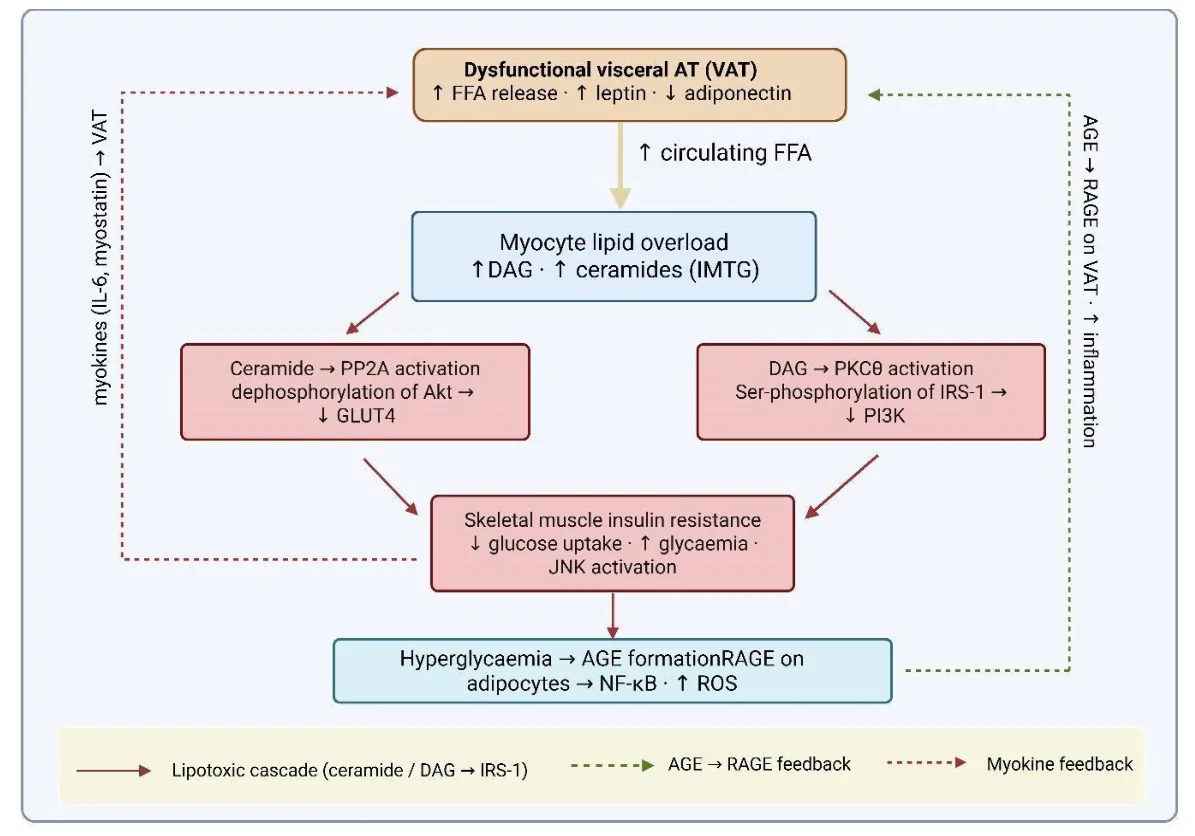
Skeletal muscle as an inflammatory–metabolic amplifier within the obesity-associated immunometabolic network. Excess lipid delivery from dysfunctional visceral adipose tissue promotes myocyte lipid overload and insulin resistance, while hyperglycaemia and advanced glycation end-products (AGEs) may further reinforce inflammatory signaling. Myokine- and AGE/RAGE-mediated feedback may contribute to a self-amplifying adipose tissue–muscle inflammatory–metabolic loop. Created in BioRender. Grudniewska, A. (2026) https://BioRender.com/phbkff8. Abbreviations: AT, adipose tissue; VAT, visceral adipose tissue; FFA, free fatty acids; DAG, diacylglycerol; IMTG, intramyocellular triglycerides; PP2A, protein phosphatase 2A; Akt, protein kinase B; GLUT4, glucose transporter type 4; PKCθ, protein kinase C theta; IRS-1, insulin receptor substrate 1; PI3K, phosphoinositide 3-kinase; JNK, c-Jun N-terminal kinase; AGE, advanced glycation end-products; RAGE, receptor for advanced glycation end-products; NF-κB, nuclear factor kappa B; ROS, reactive oxygen species; IL-6, interleukin-6.

**Figure 3:**
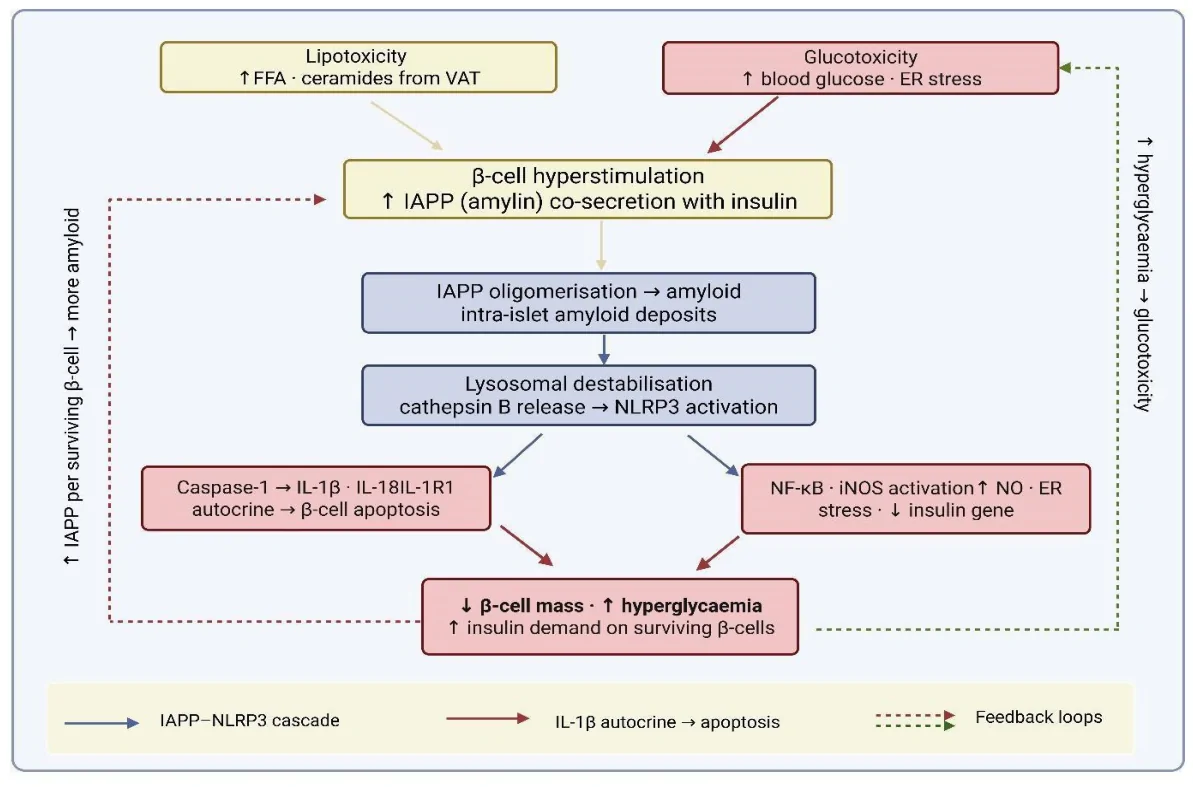
Pancreatic β-cells as a self-reinforcing inflammatory node within the obesity-associated immunometabolic network. Lipotoxicity and glucotoxicity increase metabolic stress in β-cells, promoting ER stress, mitochondrial dysfunction and ROS generation. Increased secretory demand may favour IAPP accumulation and aggregation, which can contribute to NLRP3 inflammasome activation and caspase-1-dependent maturation of IL-1β and IL-18. These processes may impair insulin production and β-cell survival, while the resulting deterioration in glycaemic control further increases metabolic stress on the remaining β-cells, contributing to a self-reinforcing cycle of β-cell dysfunction. Created in BioRender. Grudniewska, A. (2026). https://BioRender.com/sotynm3. Abbreviations: AT, adipose tissue; VAT, visceral adipose tissue; FFA, free fatty acids; ER, endoplasmic reticulum; ROS, reactive oxygen species; IAPP, islet amyloid polypeptide; NLRP3, NOD-like receptor family pyrin domain-containing 3; ASC, apoptosis-associated speck-like protein containing a CARD; CASP1, caspase-1; IL-1β, interleukin-1 beta; IL-18, interleukin-18; NF-κB, nuclear factor kappa B; iNOS, inducible nitric oxide synthase; T2DM, type 2 diabetes mellitus.

**Figure 4:**
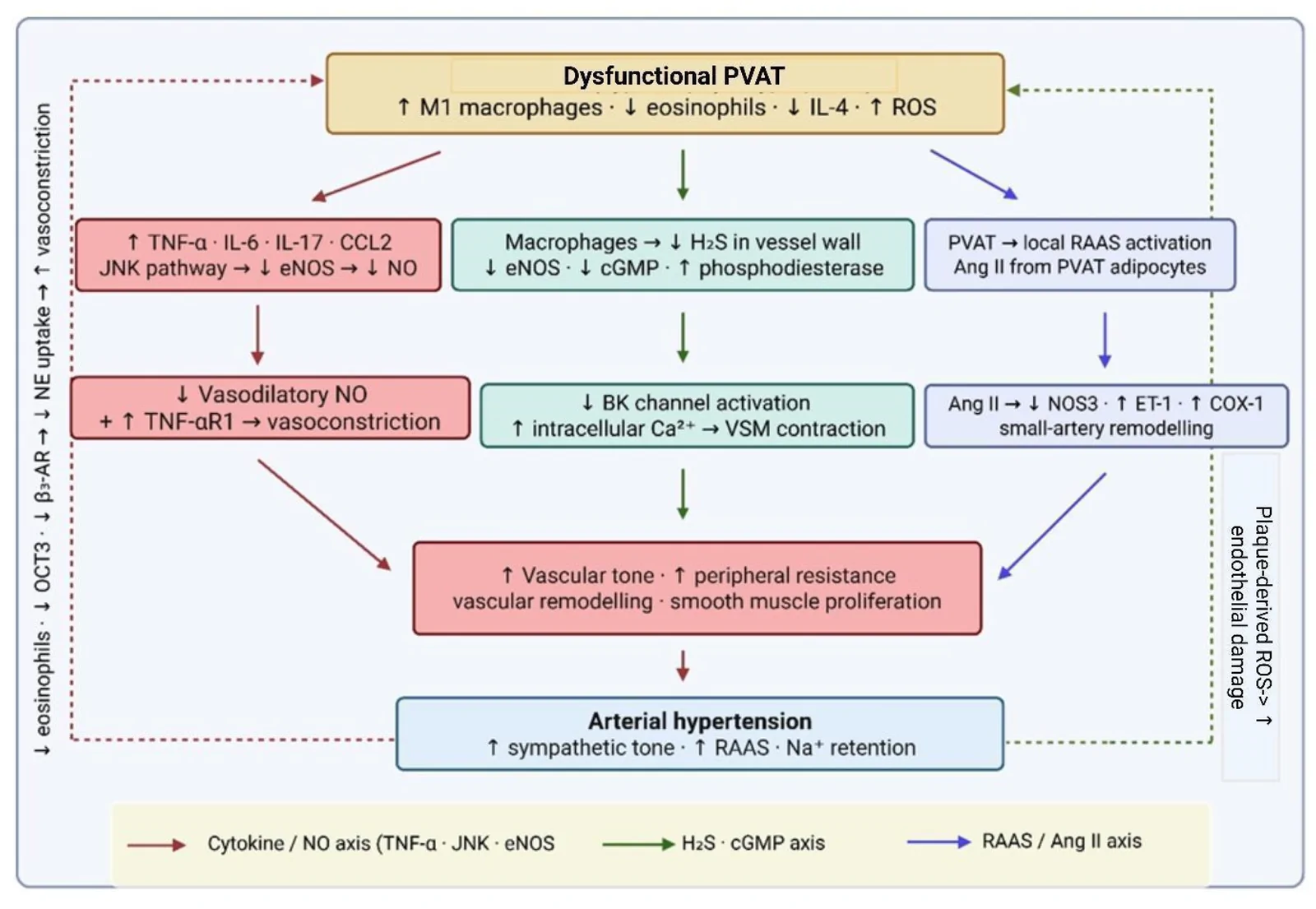
Perivascular adipose tissue dysfunction as a contributor to vascular dysfunction and arterial hypertension in obesity. Adipocyte hypertrophy and inflammatory cell infiltration alter the regulatory function of perivascular adipose tissue (PVAT), promoting endothelial dysfunction, impaired vascular relaxation and vascular remodelling. Inflammatory signalling, reduced nitric oxide and hydrogen sulfide bioavailability, and activation of the local renin–angiotensin–aldosterone system collectively increase peripheral vascular resistance and contribute to arterial hypertension. Reciprocal interactions between oxidative stress, angiotensin II and PVAT dysfunction may further reinforce this pathological cycle. Created in BioRender. Grudniewska, A. (2026). https://BioRender.com/pereo2f. Abbreviations: PVAT, perivascular adipose tissue; TNF-α, tumour necrosis factor alpha; JNK, c-Jun N-terminal kinase; eNOS, endothelial nitric oxide synthase; NO, nitric oxide; H_2_S, hydrogen sulfide; RAAS, renin–angiotensin–aldosterone system; Ang II, angiotensin II; ROS, reactive oxygen species.

**Figure 5:**
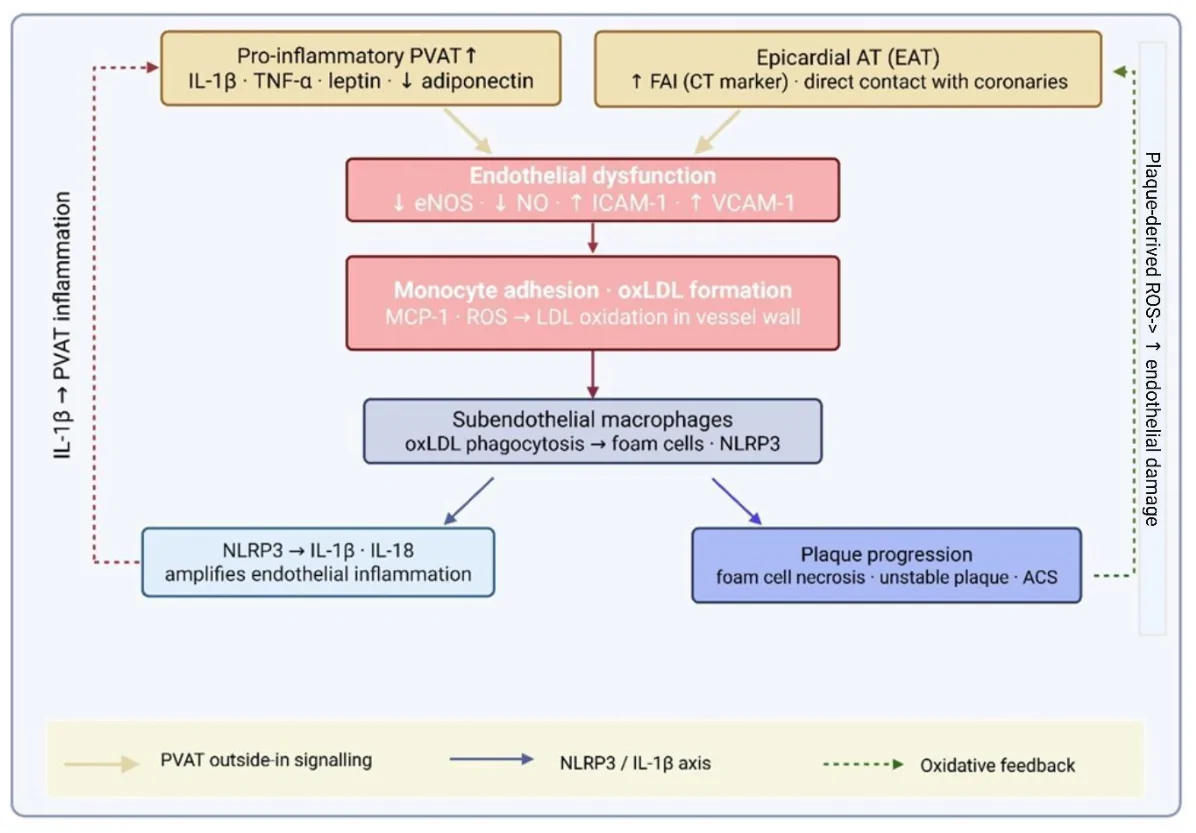
Perivascular adipose tissue and endothelial dysfunction as interconnected components of the obesity-associated vascular inflammatory network. Dysfunctional perivascular adipose tissue (PVAT) and epicardial adipose tissue (EAT) release pro-inflammatory mediators and reactive oxygen species that impair endothelial function, reduce nitric oxide bioavailability and promote vascular inflammation. Increased expression of endothelial adhesion molecules facilitates monocyte recruitment and foam cell formation, contributing to the development of an inflammatory atherosclerotic plaque. Reciprocal interactions between the endothelium, vascular plaque and dysfunctional adipose tissue may further reinforce local and systemic inflammation. Created in BioRender. Grudniewska, A. (2026). https://BioRender.com/6mtkx5j. Abbreviations: PVAT, perivascular adipose tissue; EAT, epicardial adipose tissue; FAI, fat attenuation index; IL-1β, interleukin-1 beta; IL-18, interleukin-18; TNF-α, tumour necrosis factor alpha; NF-κB, nuclear factor kappa B; eNOS, endothelial nitric oxide synthase; NO, nitric oxide; ICAM-1, intercellular adhesion molecule 1; VCAM-1, vascular cell adhesion molecule 1; oxLDL, oxidized low-density lipoprotein; NLRP3, NOD-like receptor family pyrin domain-containing 3; ROS, reactive oxygen species.

**Figure 6:**
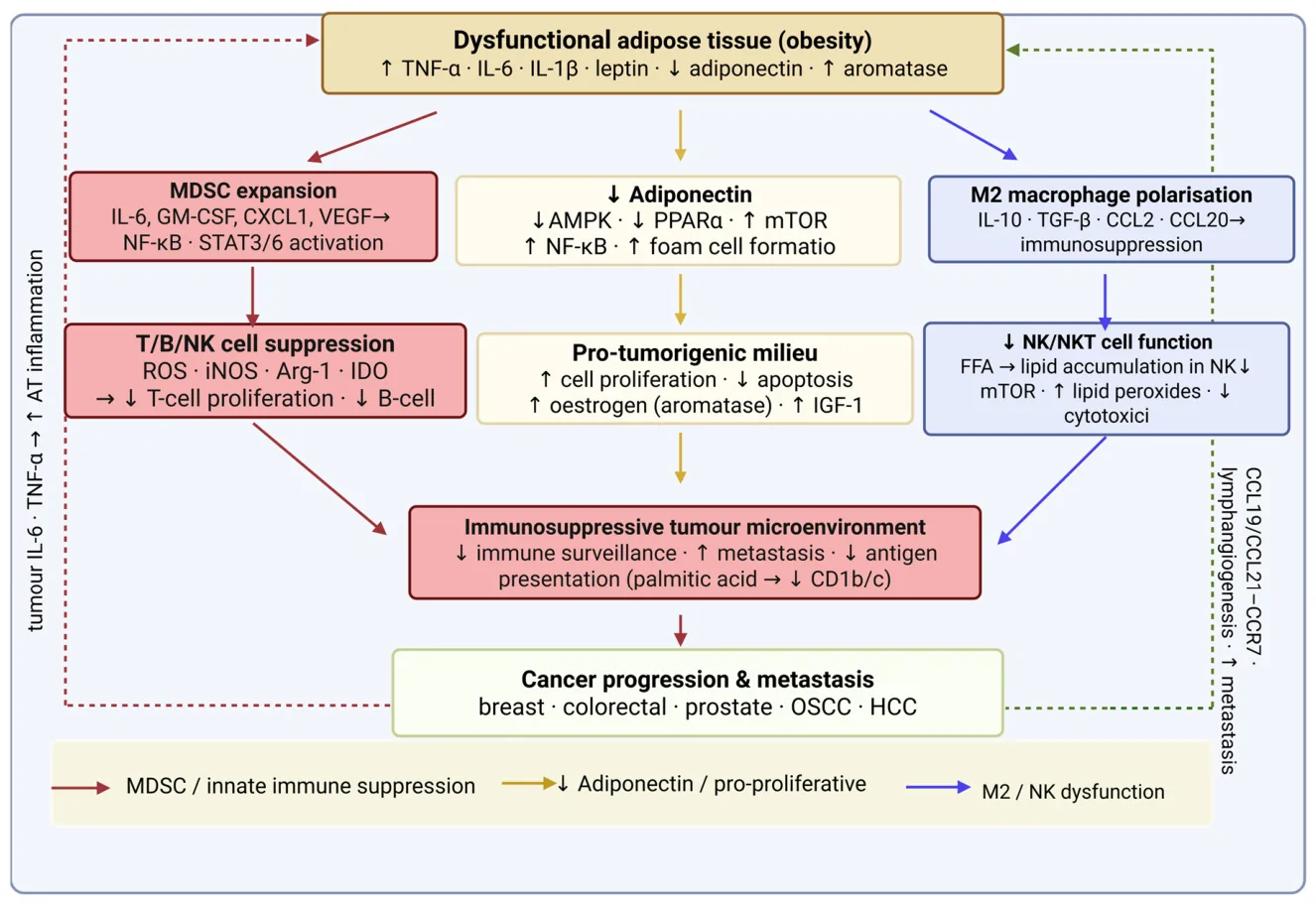
Obesity-associated immunometabolic signalling and tumour progression. Dysfunctional adipose tissue promotes a tumour-supportive environment through inflammatory signalling, adipokine imbalance and immunosuppressive myeloid-cell activity. MDSC expansion, M2 macrophage polarisation and impaired NK-cell function may weaken antitumour immune surveillance and facilitate tumour progression. Tumour-derived inflammatory signals may in turn reinforce adipose tissue dysfunction, establishing a self-reinforcing adipose tissue–tumour circuit. Created in BioRender. Grudniewska, A. (2026) https://BioRender.com/8rbjmbh. Abbreviations: AMPK, AMP-activated protein kinase; Arg-1, arginase-1; AT, adipose tissue; CCL2/19/20/21, C-C motif chemokine ligand 2/19/20/21; CCR7, C-C chemokine receptor type 7; CD1b/c, cluster of differentiation 1b/c; CXCL1, C-X-C motif chemokine ligand 1; FFA, free fatty acid; GM-CSF, granulocyte-macrophage colony-stimulating factor; HCC, hepatocellular carcinoma; IDO, indoleamine 2,3-dioxygenase; IGF-1, insulin-like growth factor 1; IL-1β/6/10, interleukin-1β/6/10; iNOS, inducible nitric oxide synthase; MDSC, myeloid-derived suppressor cell; mTOR, mechanistic target of rapamycin; NF-κB, nuclear factor κB; NK, natural killer cell; NKT, natural killer T cell; OSCC, oral squamous cell carcinoma; PPARα, peroxisome proliferator-activated receptor α; ROS, reactive oxygen species; STAT3/6, signal transducer and activator of transcription 3/6; TGF-β, transforming growth factor β; TNF-α, tumour necrosis factor α; VEGF, vascular endothelial growth factor.

**Figure 7:**
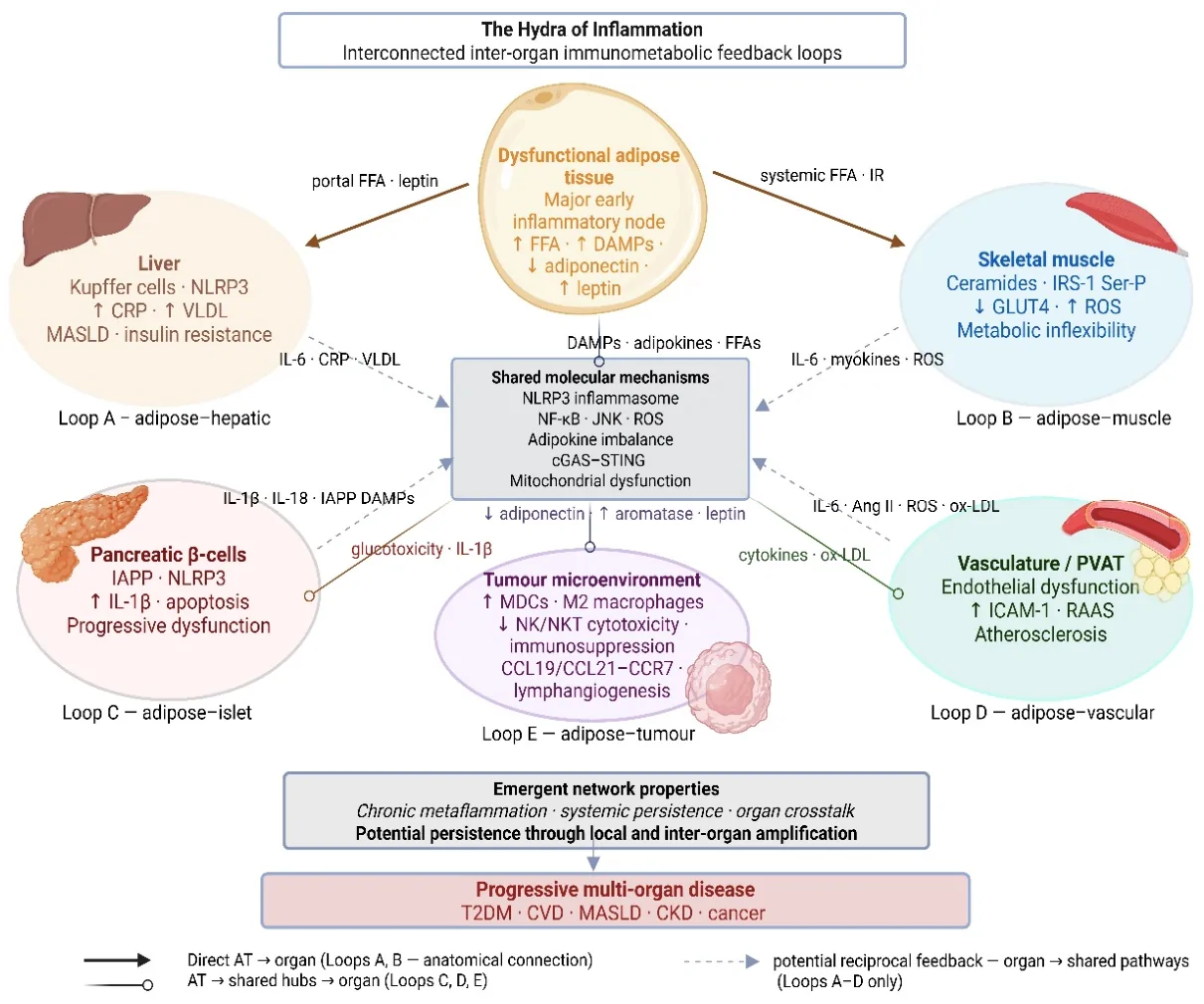
Integrated Model of the Self-Amplifying Immunometabolic Network in Obesity (The Hydra of Inflammation). Dysfunctional adipose tissue may drive five major organ-system feed-forward loops: liver (Loop A), skeletal muscle (Loop B), pancreatic β-cells (Loop C), cardiovascular system including vasculature/PVAT (Loop D), and tumour microenvironment (Loop E), through shared molecular hubs including NLRP3 inflammasome activation, NF-κB signalling, reactive oxygen species generation, adipokine imbalance and cGAS–STING pathway engagement. Once established, peripheral organ-specific loops may contribute to the persistence and reciprocal amplification of immunometabolic dysfunction, potentially reducing dependence on the initiating adipose tissue-derived signals. Together, these interconnected processes form the conceptual Hydra of Inflammation Created in BioRender. Grudniewska, A. (2026). https://BioRender.com/rwc41iw. Abbreviations. AMPK, AMP-activated protein kinase; AT, adipose tissue; CCL19/CCL21, C-C motif chemokine ligand 19/21; CCR7, C-C chemokine receptor type 7; cGAS–STING, cyclic GMP-AMP synthase–stimulator of interferon genes; CRP, C-reactive protein; DAMPs, damage-associated molecular patterns; FFA, free fatty acid; GLUT4, glucose transporter type 4; IAPP, islet amyloid polypeptide; ICAM-1, intercellular adhesion molecule 1; IL-1β/6, interleukin-1β/6; IRS-1 Ser-P, insulin receptor substrate-1 serine phosphorylation; JNK, c-Jun N-terminal kinase; MASLD, metabolic dysfunction-associated steatotic liver disease; MDSCs, myeloid-derived suppressor cells; NF-κB, nuclear factor κB; NK/NKT, natural killer/natural killer T cell; NLRP3, NOD-, LRR- and pyrin domain-containing protein 3; ox-LDL, oxidised low-density lipoprotein; PVAT, perivascular adipose tissue; RAAS, renin–angiotensin–aldosterone system; ROS, reactive oxygen species; TNF-α, tumour necrosis factor α; VLDL, very low-density lipoprotein.

**Table 1:** Main inflammatory pathways, affected organs, metabolic consequences, and type of supporting evidence in obesity-associated immunometabolic disease. ↑ indicates an increase in hormone concentration; ↓ indicates a decrease in hormone concentration.

Pathway/Mediator	Affected Organs	Metabolic Consequence	Type of Evidence	References
NF-κB activation	Adipose tissue, liver, skeletal muscle, pancreas, endothelium	Pro-inflammatory cytokine production (IL-6, TNF-α, IL-1β); insulin resistance; endothelial dysfunction	Experimental + human translational	[3,7,8,10]
NLRP3 inflammasome/IL-1β	Adipose tissue, liver, pancreatic β-cells, skeletal muscle	IL-1β/IL-18 secretion; β-cell apoptosis; hepatic steatosis amplification; peripheral IR	Experimental + clinical (T2D)	[15,16,17,26,27,29]
JNK pathway	Hepatocytes, myocytes, adipocytes	IRS-1 serine phosphorylation; impaired insulin signalling; hepatic gluconeogenesis	Experimental + human	[7,8,23]
Adipokine imbalance (↓ adiponectin, ↑ leptin)	Liver, skeletal muscle, cardiovascular system, tumour microenvironment	Reduced insulin sensitisation; increased inflammation; impaired NK cell function	Experimental + translational	[14,28,54,55]
Lipotoxicity (FFAs, DAG, ceramides)	Liver, skeletal muscle, pancreatic β-cells, PVAT, endothelium	ER and mitochondrial stress; ROS; insulin signal disruption; lipoapoptosis	Experimental + human histology	[3,5,6,19,23,25]
PVAT/EAT dysfunction	Coronary and peripheral arteries, endothelium	Vasoconstriction; endothelial NF-κB activation; atherosclerotic plaque progression	Experimental + translational	[32,33,34,35,36]
cGAS–STING pathway	Adipose tissue, liver	Type I interferon; NLRP3 priming; perpetuation of sterile inflammation	Predominantly experimental	[70,71]
MDSC expansion	Tumour microenvironment, systemic	Suppression of T-cell and NK-cell antitumour responses; cancer progression	Experimental + limited human	[51,52,58,59,60]

## Data Availability

No new data were created or analysed in this study. Data sharing is not applicable to this article.

## Abbreviations

The following abbreviations are used in this manuscript: ADMA, asymmetric dimethylarginine; AGE, advanced glycation end product; Akt, protein kinase B; AMPK, AMP-activated protein kinase; Arg-1, arginase-1; AT, adipose tissue; CCL, C-C motif chemokine ligand; CCR, C-C chemokine receptor; cGAS, cyclic GMP-AMP synthase; CRP, C-reactive protein; DAG, diacylglycerol; DAMPs, damage-associated molecular patterns; EAT, epicardial adipose tissue; ECs, endothelial cells; eNOS, endothelial nitric oxide synthase; FAI, fat attenuation index; FFA, free fatty acid; GLUT4, glucose transporter type 4; H_2_S, hydrogen sulfide; IAPP, islet amyloid polypeptide; ICAM-1, intercellular adhesion molecule 1; IDO, indoleamine 2,3-dioxygenase; IGF-1, insulin-like growth factor 1; IL, interleukin; iNOS, inducible nitric oxide synthase; IR, insulin resistance; IRS-1, insulin receptor substrate 1; JNK, c-Jun N-terminal kinase; MASLD, metabolic dysfunction-associated steatotic liver disease; MASH, metabolic dysfunction-associated steatohepatitis; MDSCs, myeloid-derived suppressor cells; MMP, matrix metalloproteinase; mTOR, mechanistic target of rapamycin; NF-κB, nuclear factor kappa B; NK, natural killer; NKT, natural killer T cell; NLRP3, NOD-, LRR- and pyrin domain-containing protein 3; NO, nitric oxide; OCT3, organic cation transporter 3; oxLDL, oxidized low-density lipoprotein; PAI-1, plasminogen activator inhibitor 1; PI3K, phosphoinositide 3-kinase; PKC, protein kinase C; PPAR, peroxisome proliferator-activated receptor; PVAT, perivascular adipose tissue; RAAS, renin–angiotensin–aldosterone system; RAGE, receptor for advanced glycation end products; ROS, reactive oxygen species; SAA, serum amyloid A; STAT, signal transducer and activator of transcription; STING, stimulator of interferon genes; TGF-β, transforming growth factor beta; TLR4, Toll-like receptor 4; TNF-α, tumour necrosis factor alpha; VCAM-1, vascular cell adhesion molecule 1; VLDL, very low-density lipoprotein.
